# A benchmark dataset for canopy crown detection and delineation in co-registered airborne RGB, LiDAR and hyperspectral imagery from the National Ecological Observation Network

**DOI:** 10.1371/journal.pcbi.1009180

**Published:** 2021-07-02

**Authors:** Ben G. Weinstein, Sarah J. Graves, Sergio Marconi, Aditya Singh, Alina Zare, Dylan Stewart, Stephanie A. Bohlman, Ethan P. White

**Affiliations:** 1 Department of Wildlife Ecology and Conservation, University of Florida, Gainesville, Florida, United States of America; 2 Nelson Institute for Environmental Studies, University of Wisconsin-Madison, Madison, Wisconsin, United States of America; 3 Department of Agricultural & Biological Engineering, University of Florida, Gainesville, Florida, United States of America; 4 Department of Electrical and Computer Engineering, University of Florida, Gainesville, Florida, United States of America; 5 School of Forest Resources and Conservation, University of Florida, Gainesville, Florida, United States of America; 6 Informatics Institute, University of Florida, Gainesville, Florida, United States of America; 7 Biodiversity Institute, University of Florida, Gainesville, Florida, United States of America; Abdus Salam International Centre for Theoretical Physics, ITALY

## Abstract

Broad scale remote sensing promises to build forest inventories at unprecedented scales. A crucial step in this process is to associate sensor data into individual crowns. While dozens of crown detection algorithms have been proposed, their performance is typically not compared based on standard data or evaluation metrics. There is a need for a benchmark dataset to minimize differences in reported results as well as support evaluation of algorithms across a broad range of forest types. Combining RGB, LiDAR and hyperspectral sensor data from the USA National Ecological Observatory Network’s Airborne Observation Platform with multiple types of evaluation data, we created a benchmark dataset to assess crown detection and delineation methods for canopy trees covering dominant forest types in the United States. This benchmark dataset includes an R package to standardize evaluation metrics and simplify comparisons between methods. The benchmark dataset contains over 6,000 image-annotated crowns, 400 field-annotated crowns, and 3,000 canopy stem points from a wide range of forest types. In addition, we include over 10,000 training crowns for optional use. We discuss the different evaluation data sources and assess the accuracy of the image-annotated crowns by comparing annotations among multiple annotators as well as overlapping field-annotated crowns. We provide an example submission and score for an open-source algorithm that can serve as a baseline for future methods.

## Author summary

Combining RGB, LiDAR and hyperspectral sensor data from the USA National Ecological Observatory Network’s Airborne Observation Platform with multiple types of evaluation data, we created a benchmark dataset to assess crown detection and delineation methods for canopy trees covering dominant forest types in the United States. This benchmark dataset includes an R package to standardize evaluation metrics and simplify comparisons between methods. The benchmark dataset contains over 6,000 image-annotated crowns, 400 field-annotated crowns, and 3,000 canopy stem points from a wide range of forest types.

This is a *PLOS Computational Biology* Benchmarking paper.

## Introduction

Quantifying individual trees is a central task for ecology and management of forested landscapes. Compared to traditional field surveys, airborne remote sensing allows forest monitoring at broad scales. A central task in remote sensing of forests is converting raw sensor data into information on individual trees [1]. While there are dozens of proposed algorithms, they are often designed and evaluated using a range of different data inputs [2–4], sensor resolutions, forest structures, evaluation protocols [5–8], and output formats [9]. For example, [10] proposed a pixel-based algorithm for 50 cm pan-sharpened satellite RGB data from a tropical forest in Brazil evaluated against field-collected tree stem locations, and [11] proposed a vector-based algorithm for 10 cm fixed-winged aircraft RGB data from oak forests in California evaluated against image-annotated crowns. Given these differences, a comparison among algorithms is difficult to make based on reported statistics to interpret the relative accuracy, generality and cost effectiveness.

One solution to these challenges is a benchmark dataset that can be used to evaluate a wide variety of algorithms and data types [12, 13]. We believe a useful benchmark dataset has at least three features [13–16]: 1) well-curated and open-source data, 2) reasonable evaluation criteria, 3) reproducible and transparent scoring. We developed a benchmark dataset of individual canopy crowns derived from multi-sensor imagery in the National Ecological Observatory Network (Table 1) that provides: 1) co-registered remote sensing data from multiple sensors (LiDAR, RGB imagery, and hyperspectral imagery) to allow comparisons of methods based on any single sensor (e.g., for LiDAR based methods), or any combination of sensors (e.g., combining RGB and hyperspectral), and 2) three types of evaluation data to allow assessing both ‘tree detection’, defined as the identifying the location of individual trees using evaluation data with a point at the crown center [5, 17], and ‘crown delineation’ defined as identifying the boundary edge of crowns [9, 11–13] across a broad range of forest types. The benchmark is designed to allow flexibility in both workflow and sensor selection. Users of the benchmark can use any combination of algorithms and sensors so long as the final product is a 2-dimensional shape with geographic coordinates representing the boundaries of individual canopy tree crowns.

**Table 1:** Summary of datasets included in the benchmark dataset. All sensor data has been cropped to the extent of NEON field sampling plots.

Item (format)	Type	Description (NEON ID)
10cm RGB data (.tif)	Sensor data	DP3.30010.001
LiDAR point cloud (~5 pts/m) (.laz)	Sensor data	DP1.30003.001
1m gridded raster of canopy height model (.tif)	Sensor data	DP3.30015.001
1m 426 band hyperspectral data	Sensor data	DP1.30006.001
Image-annotated crowns (.xml)	Evaluation data (6490 trees)	Bounding box annotations made by visually assessing the sensor data
Field-annotated crowns (.shp)	Evaluation data (562 trees)	Polygon annotations by visually assessing the hyperspectral data while physically in the field next to target tree
Field-collected stems (.csv)	Evaluation data (4365 trees)	NEON collected stem points for each individual tree. Filtered from the Woody Vegetation Structure data product (NEON ID: DP1.10098.001)

## Methods and results

### Remote sensing data

The National Ecological Observatory Network (NEON) is a large initiative to coordinate data collection across the United States at over 80 geographic sites. Annual data collection includes surveys by the airborne observation platform (AOP) using RGB, LiDAR and hyperspectral sensors (http://data.neonscience.org/), as well as standardized 40m vegetation surveys at fixed sampling plots throughout each site. The NEON AOP uses fixed-wing aircraft, flown around 1000m above ground, to survey sites during leaf-on-conditions from May-October. Sensor data chosen for this benchmark were collected during flights from 2018 and 2019. For the purposes of the benchmark dataset, we cropped sensor products to the bounds of each 40m NEON field sampling plot. For example, the RGB image ‘SJER_052_2019’ corresponds to NEON field plot 52 at NEON site SJER (San Joaquin, California see Table 2 for abbreviations) with sensor data from the 2019 airborne survey. For additional detail on NEON design and planning, see NEON’s extensive technical documents for detailed site information and sampling strategy (neonscience.org).

**Table 2:** Annotations for each data type for each of the NEON sites.

siteID	Site Name	State	Image-annotated Evaluation Crowns	Field-collected Stems	Additional data or notes
ABBY	Abby Road	WA	160	14	
BART	Bartlett Experimental Forest	NH	93	535	369 image-annotated training crowns
BLAN	Blandy Experimental Farm	VA	73	0	
BONA	Caribou-Poker Creeks Research Watershed	AK	225	0	
CLBJ	Lyndon B. Johnson National Grassland	TX	116	0	
DEJU	Delta Junction	AK	0	60	
DELA	Dead Lake	AL	87	240	295 image-annotated training crowns
DSNY	Disney Wilderness Preserve	FL	87	0	888 image-annotated training crowns
HARV	Harvard Forest	MA	171	622	329 image-annotated training crowns
JERC	The Jones Center At Ichauway	GA	294	159	
LENO	Lenoir Landing	AL	75	103	554 image-annotated training crowns
MLBS	Mountain Lake Biological Station	VA	481	668	1921 image-annotated training crowns, 106 field-annotated crowns
MOAB	Moab	UT	0	11	
NIWO	Niwot Ridge	CO	1485	500	10,022 image-annotated training crowns
ONAQ	Onaqui	UT	32	0	244 image-annotated training crowns
OSBS	Ordway-Swisher Biological Station	FL	497	346	2126 image-annotated training crowns, 458 field-annotated crowns
SCBI	Smithsonian Conservation Biology Institute	VA	73	193	
SERC	Smithsonian Environmental Research Center	MD	94	369	
SJER	San Joaquin Experimental Range	CA	473	57	2545 image-annotated training crowns
SOAP	Soaproot Saddle	CA	114	0	
TALL	Talladega National Forest	AL	157	220	
TEAK	Lower Teakettle	CA	1471	0	1471 image-annotated training crowns
UKFS	University of Kansas Field Station	KS	0	127	
UNDE	University of Notre Dame Environmental Research Center	MI	186	66	
WREF	Wind River Experimental Forest	WA	178	0	
YELL	Yellowstone National Park	WY	0	0	873 image-annotated training crowns

### Orthorectified camera mosaic

**Fig 1:**
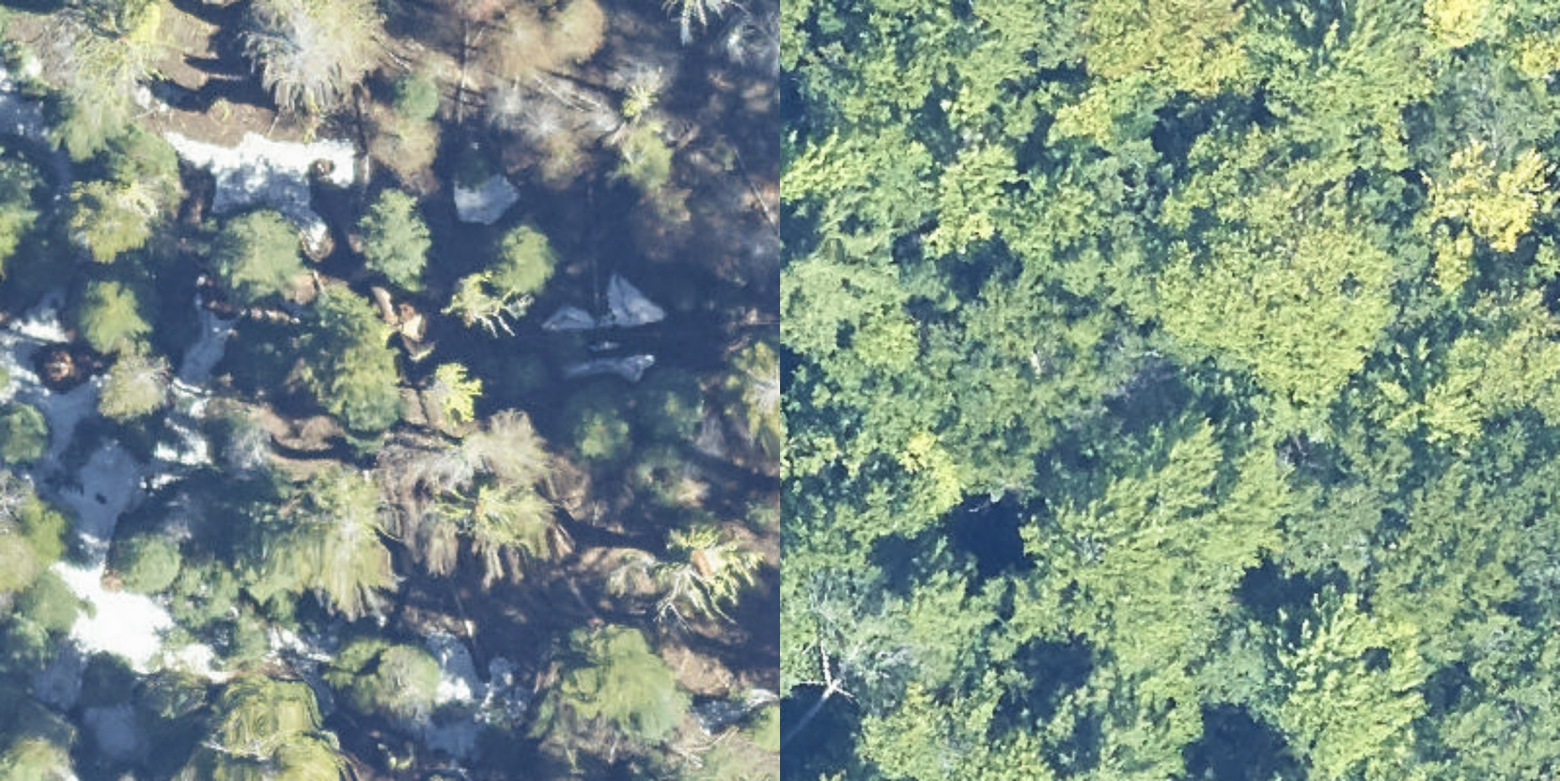
A 40m x 40m evaluation plot of RGB data from the Teakettle Canyon (TEAK) NEON site (left) and Bartlett Experimental Forest, New Hampshire (BART) (right).

The RGB data were acquired with a D8900 camera with a format of 8,984 x 6,732 pixels. Individual images were color rectified, orthorectified and mosaiced to create a single raster image with a pixel size of 0.1 m^2. Mosaic tiles are provided as 1000m x 1000m geoTIFF files and are named based on the UTM coordinate at the northwest origin. RGB data have high spatial resolution and individual canopy trees are often visible based on the crown boundary, as well as color differences among individuals due to taxonomy and health status (Fig 1). For more details on NEON camera orthomosaic products see NEON technical document NEON.DOC.005052 [18].

One challenge in creating a multi-sensor dataset is the joint georectification of data types. To ensure spatial overlap between the LiDAR and RGB data, NEON staff overlaid the 0.1m spatial resolution RGB tile on a 1m spatial resolution LiDAR derived surface height model. The difference in spatial resolution can cause some distortion in rectified RGB images. These artifacts are most pronounced at the image edge and were minimized by selecting the centermost portion of each image when creating the RGB mosaic. Some distortion remains and can cause a swirling effect as the image pixels are stretched to match the corresponding LiDAR raster cell. For more information see NEON technical document NEON.DOC.001211vA [18]. We did not include images with large enough distortions to interfere with canopy crown detection but kept images with minor distortions to represent the kind of challenging conditions present in applied settings.

### Classified LiDAR Point Cloud

**Fig 2:**
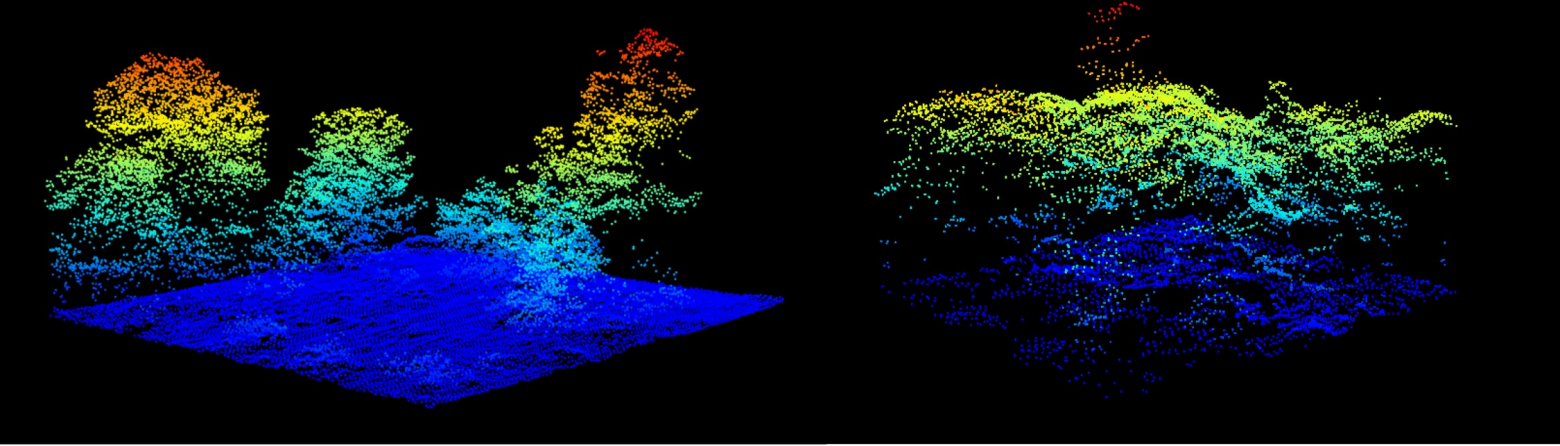
Normalized LIDAR point cloud for evaluation plot SJER_064 from the San Joaquin Experimental Range, California (left) and MLBS_071 from Mountain Lake Biological Station, Virginia. Points are colored by height above ground.

The LiDAR data are 3D coordinates (~5 points/m^2^) that provide high resolution information about canopy crown shape and height. LiDAR data are stored as 1000m x 1000m.laz files (Fig 2). These files contain the x,y,z coordinates for each return, as well as metadata on return intensity and point classification. Boundaries of individual canopy crowns are often apparent due to gaps among neighboring trees or differences in height among overlapping canopy crowns. For more information on NEON LiDAR data processing see NEON technical document NEON.DOC.001292 [19]. Due to the large spatial coverage of the collection effort, the point density of the NEON LiDAR clouds is much lower than the point density used for most studies of crown detection models ([20, 21]; point densities of 8–1000 pt/m^2^).

### Hyperspectral surface reflectance

**Fig 3:**
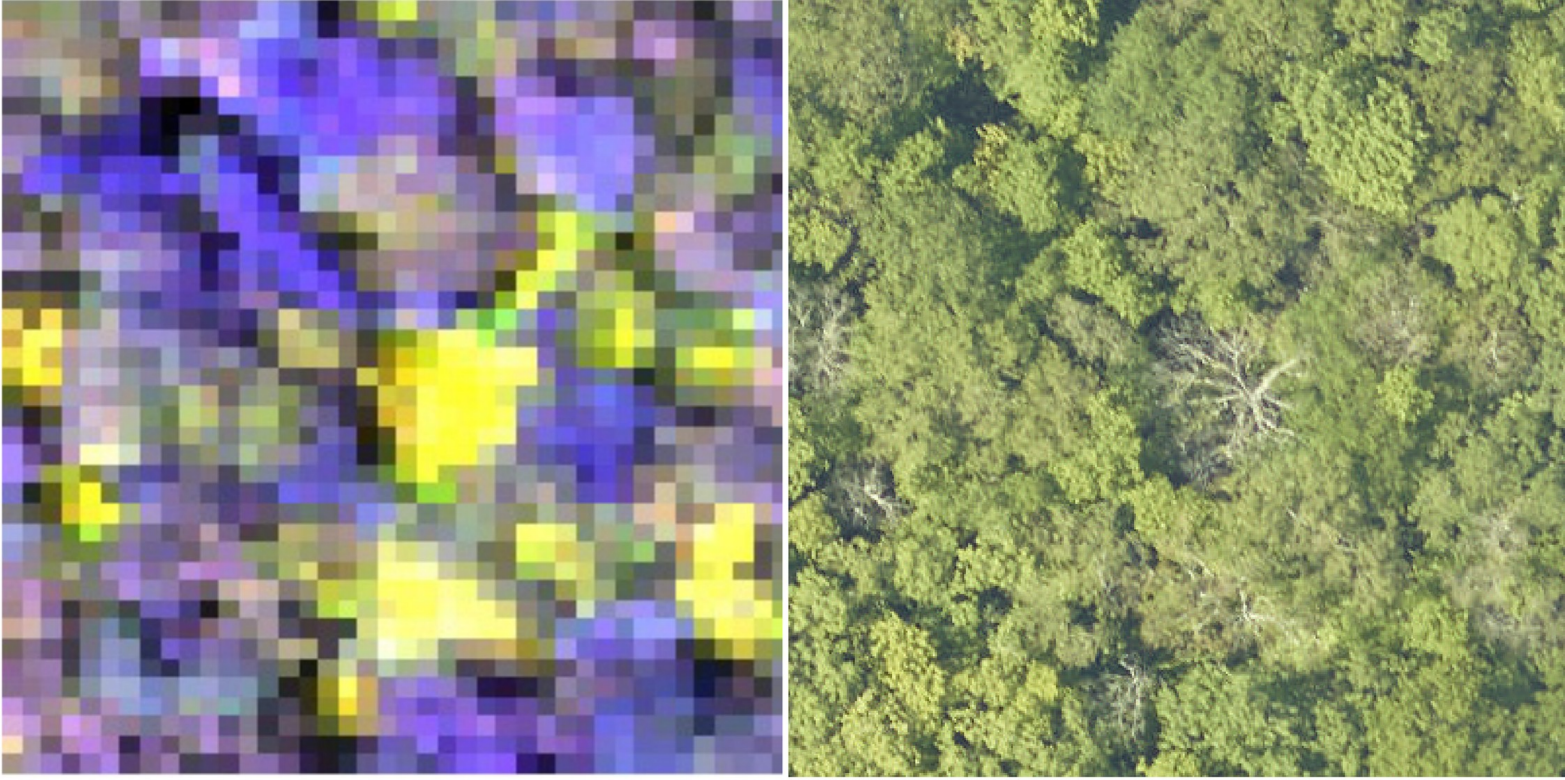
Composite hyperspectral image (left) and corresponding RGB image (right) for the MLBS site. The composite image contains near infrared (940nm), red (650nm), and blue (430nm) channels. Trees that are difficult to segment in RGB imagery may be more separable in hyperspectral imagery due to the differing foliar chemical and structural properties of co-occurring trees.

NEON’s hyperspectral sensor collects reflected light in the visible and infrared spectrum between approximately 420–2500 nm with a spectral sampling interval of 5nm for a total of 426 bands. NEON provides the orthorectified images with a pixel size of 1 m^2^ in 1000m x 1000m tiles that align with the RGB and LiDAR file naming convention. Hyperspectral data, especially in the infrared spectrum, is often used for differentiating tree species based on spectral differences among species in leaf chemistry and canopy structure [22]. Hyperspectral data is particularly useful in forests with high species diversity where neighboring trees are likely to be different species and thus spectrally distinct (Fig 3)[23]. All hyperspectral data were collected during the same field collection campaign as the RGB data, with the exception of the UNDE site, in which the 2019 RGB data was not available at the time of publication and therefore the 2017 flight data was used instead. For more information on hyperspectral data processing and calibration see NEON technical document NEON.DOC.001288 [24].

### Ecosystem structure

NEON’s ‘Ecosystem Structure’ data product is a LiDAR derived height raster at 1m spatial resolution. Often known as a ‘canopy height model’ (CHM), the raster values are the normalized height above ground for each grid cell. This data is useful for differentiating crowns in three dimensions, as well as eliminating crowns that are under the 3m threshold used in this benchmark for minimum tree height. For more information on normalization and interpolation to create the raster product see NEON technical document NEON.DOC.002387 [25].

### Woody plant vegetation structure

Along with sensor data, NEON collects information on trees in fixed plots at each NEON site. Data from two types of plots are included in this dataset: ‘distributed’ plots, which are 20m x 20m fully sampled plots, and ‘Tower’ plots, which are 40m x 40m plots with two sampled 20m x 20m quadrants. The distinction between distributed and tower plots may be useful for users familiar with NEON’s sampling regime, but is not necessary for most uses of the benchmark data set. All trees in sampled areas with a stem diameter of > 10cm are mapped and recorded. For the purposes of this benchmark dataset, the key tree metadata are the stem position, size, and estimated tree height. For extensive information on NEON field sampling see NEON technical document NEON.DOC.000987 [26].

### Evaluation annotations

The goal of this benchmark is to evaluate algorithms for canopy tree detection and delineation. We adopt the term ‘canopy crown detection’ to differentiate between the tasks of ‘tree detection’, defined as identifying the location of the crown center of individual trees [5, 17] and ‘crown delineation’ or ‘crown segmentation’, often defined as identifying the boundary edge of individual crowns [9, 27–29]. The term ‘canopy’ is often implicitly assumed in most studies, since optical data and low density LiDAR data, can only reflect the structure in the upper canopy ([30] but see [31, 32]). Evaluation of detection methods in this benchmark dataset is done by assessing detections using three types of evaluation data: 1) image-annotated crown bounding boxes for 22 sites in the NEON network, 2) field-annotated crown polygons for two sites in the NEON network (Table 2), and 3) field-collected stem points from 14 sites from the NEON Woody Vegetation Structure dataset. For each of these data we outline how the data were collected and the evaluation procedure for canopy crown detection.

### Image-annotated crowns

**Fig 4:**
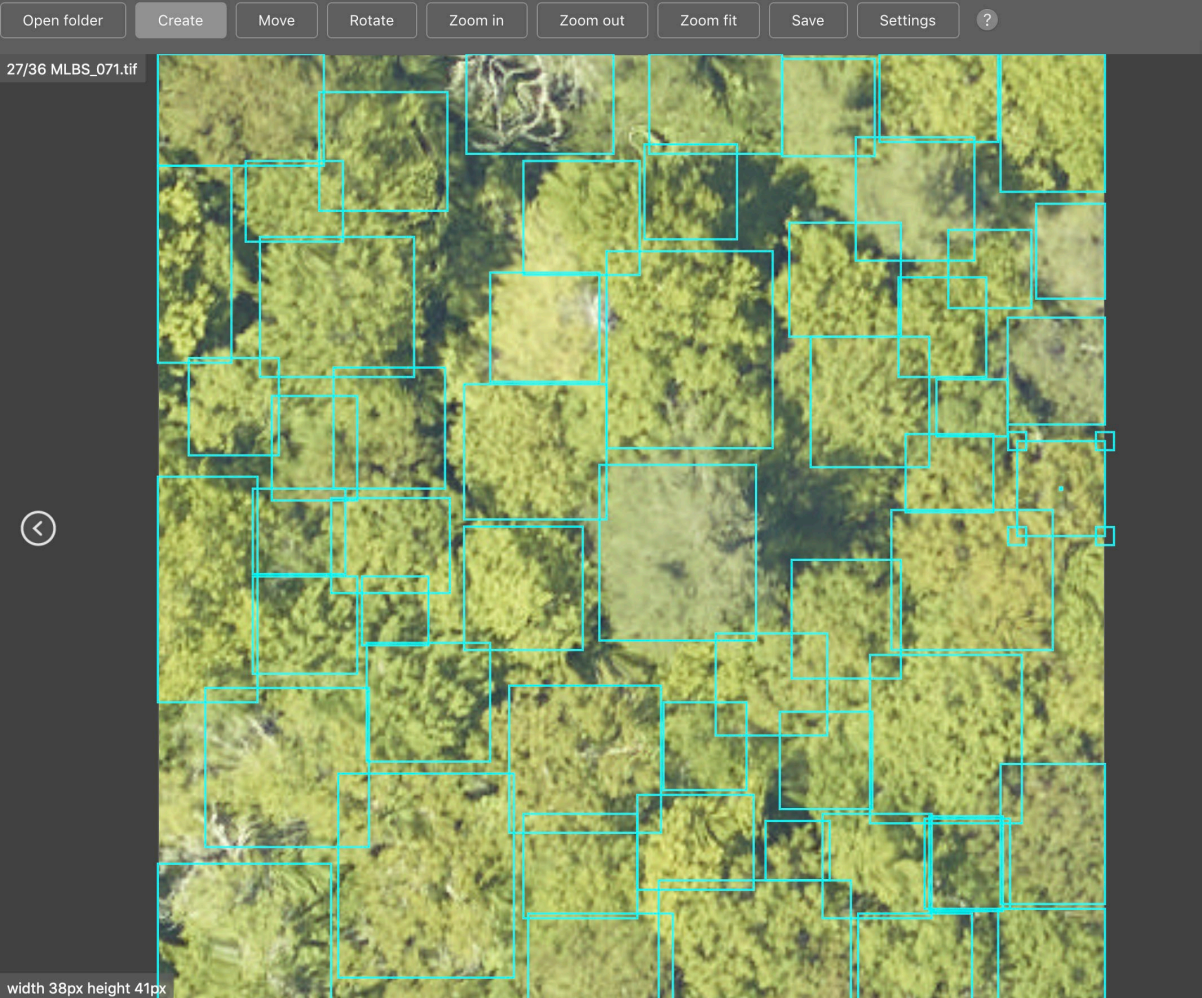
Screenshot of the program RectLabel used for tree annotation for the image-annotated crowns for NEON plot MLBS_071. For each visible tree crown, we created a four point bounding box.

We selected airborne imagery from 22 sites surveyed by the NEON AOP. The evaluation sites were chosen based on the availability of the three types of sensor data, as well as representation of forest conditions across the US including the diversity of species composition, stand age, and canopy openness. The selected sites range from Florida to Alaska, include forest types dominated by conifers, broadleaves or a mixture of the two, and varying in density from open oak woodlands (3.5 trees per 20m plot at the SJER site) to dense deciduous forests (34.38 trees per plot at the HARV site). Images were annotated using the program RectLabel (Table 1). For each visible tree, we created a bounding box (xmin, ymin, xmax, ymax) that covered the tree crown (Fig 4). We prefer bounding boxes over polygons for image-annotated crowns for speed of annotation, which is needed to cover the large number of images and sites to make a benchmark on geographic generalization possible.

**Fig 5:**
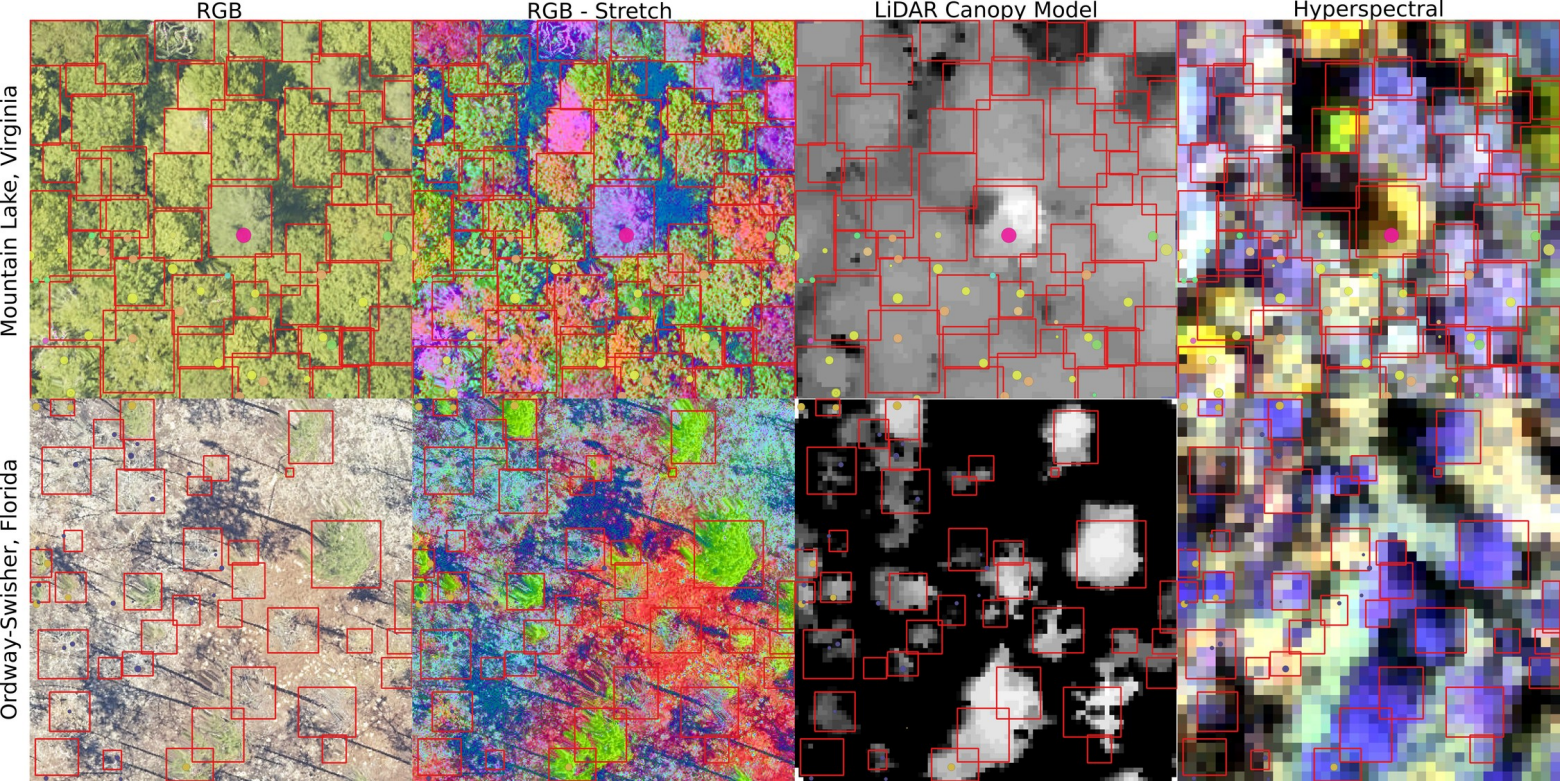
Image-annotated tree crowns for the evaluation data set for two sites in the National Ecological Observation Network. Using the RGB, LiDAR and hyperspectral products together contributes to more careful crown annotation. For some sites, such as MLBS (top row), the RGB and hyperspectral data are useful for differentiating overlapping crowns. For other sites, such as OSBS (bottom row) the LiDAR point cloud, shown as a rasterized height image, is most useful in capturing crown extent. The RGB-stretch image was produced by transforming the RGB data in the three principal components space. To create a three-band hyperspectral image, we used channels from the red, blue and infrared spectrum to capture changes in reflectance not apparent in the RGB imagery.

We carefully annotated the evaluation images by comparing the RGB, LiDAR and hyperspectral data. Using all three products made it possible to more accurately distinguish neighboring trees in images by simultaneously assessing visual patterns (RGB), using variation in spectral signatures to distinguish different species (hyperspectral), and looking at the three-dimensional structure of the tree (LiDAR). For some sites, such as OSBS, the crowns were most visible in the LiDAR height model, whereas for closed canopy sites such as MLBS, the hyperspectral and RGB data were most useful. When working with the hyperspectral data we primarily used a composite three-band hyperspectral image containing near infrared (940nm), red (650nm), and blue (430nm) channels, which showed contrasts between neighboring trees of different types (Fig 5D and 5H). We also augmented the RGB data to view subtle changes in pixel values using a decorrelation stretch (Fig 5B and 5F). The decorrelation stretch is useful in highlighting small differences within the image color space that are not apparent in the visual RGB color spectrum. Each evaluation plot overlaps with a NEON 40m x 40m plot. Within each of these plots, NEON field crews survey a 20x20 subplot; therefore, while field data are available for most plots in the dataset, they do not cover every tree in the image. The woody vegetation structure data contains information on field estimated height and maximum crown diameter for the majority of field collected stems. We annotated all trees in the 40x40 m plot, regardless of health status, provided they were visible in the image.

### Field-annotated crowns

Individual trees were annotated by visiting two NEON sites and mapping the tree crown boundaries as polygons in the remote sensing images using a field tablet and GIS software while looking at each tree from the ground [33]. False-color composites from the hyperspectral data, RGB, and LiDAR canopy height model were loaded onto tablet computers that were equipped with GPS receivers. While in the field, researchers digitized crown boundaries based on the location, size, and shape of the crown. Only alive trees with leaf-on vegetation were selected. Trees were mapped in 2014 and 2015, and all polygons were manually checked against the most recent NEON imagery. All crowns that were no longer apparent in the RGB or LiDAR data due to tree fall or overgrowth were removed from the dataset, and minor adjustments to crown shape and position were refined after examining multiple years of RGB imagery. No adjustments to the polygons were made due to crown expansion.

### Evaluation for image-annotated and field-annotated crowns

**Fig 6:**
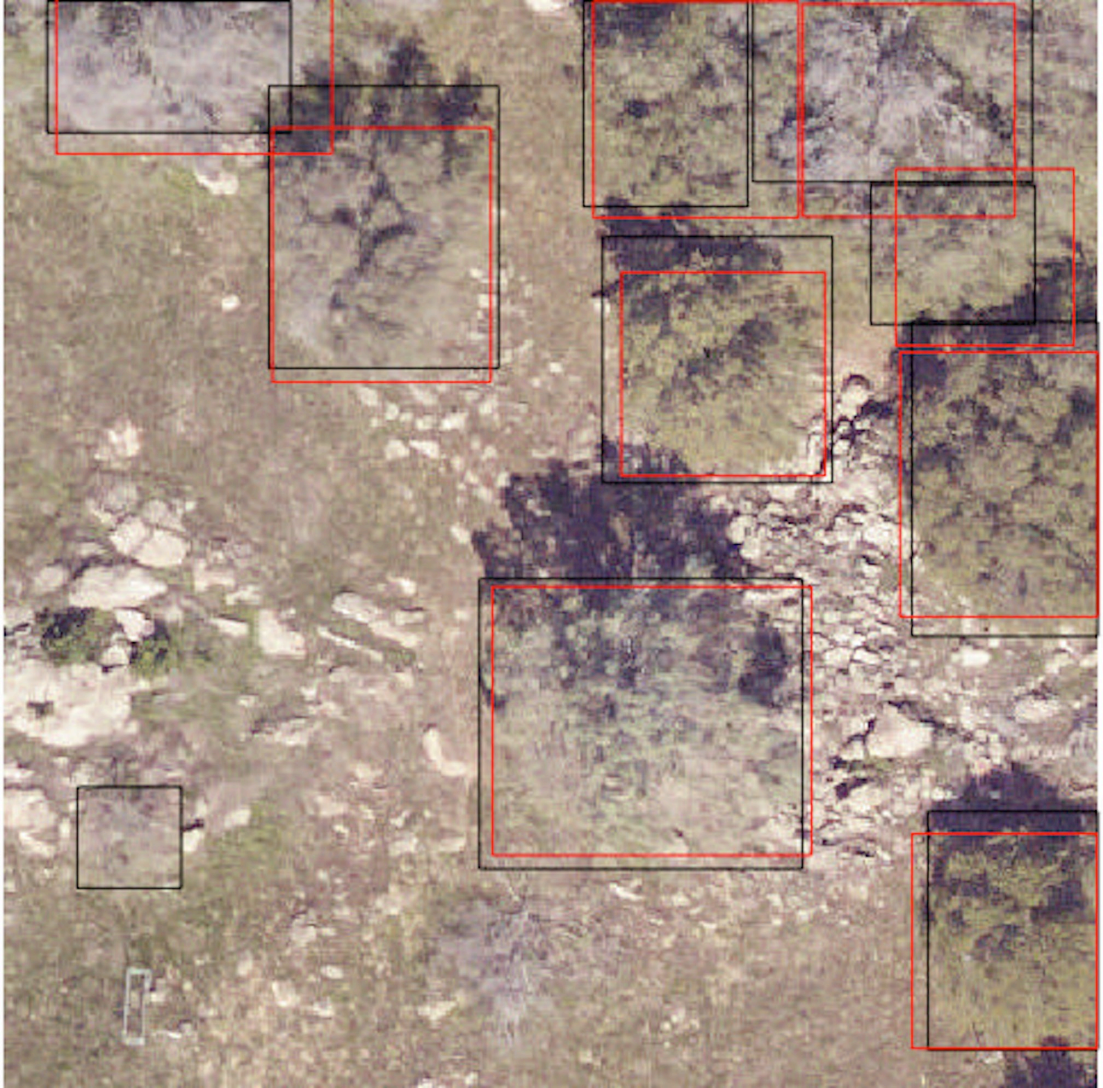
Example evaluation from the NeonTreeEvaluation R package. Predicted boxes (see below) in red and ground truth boxes are in black. In this image there are 10 image-annotated boxes, and 9 predictions. Each prediction matches an image-annotated box with an intersection-over-union score of greater than 0.4. This leads to a recall score of 0.9 and a precision score of 1.

The evaluation procedure in this benchmark is identical for image-annotated and field annotated crowns, since the final data format for both is a geospatial file with either bounding boxes (image-annotated) or polygons (field-annotated) for each canopy crown. To measure accuracy and precision of predicted detections, the most common approach is to compare the overlap between predicted crowns and evaluation crowns using the intersection-over-union metric (IoU; e.g.[34]) and a minimum matching threshold. IoU is the area of the overlap between the predicted crown and the evaluation crown divided by the area of the combined region. Any comparisons with a IoU score above the minimum threshold are true positives. The metric ranges between 0 (no overlap) to 1 (perfect overlap) (Fig 6). In the wider computer vision literature, the conventional threshold value for true positive overlap is 0.5 (e.g.[34]), but this value is arbitrary and does not ultimately relate to any particular ecological question. We tested a range of overlap thresholds from 0.3 (less overlap among matching crowns) to 0.6 (more overlap among matching crowns) and found that 0.4 balanced a rigorous cutoff without spuriously removing trees that would be useful for downstream analysis. Using this overlap threshold, the benchmark code calculates recall, defined as the proportion of crowns correctly predicted, and precision, defined as the proportion of predictions that matched a ground truth crown. If multiple predictions overlap a single ground truth crown, we match the prediction with the highest IoU to the ground truth. Predictions that do not overlap with any ground truth are considered false positives. To create a single summary statistic for the entire benchmark, we calculate the mean precision and recall per image rather than pooling results across sites. We chose this statistic to emphasize the wide geographic variance in forest types.

### Field-collected stems

NEON field crews sample all trees within a plot that are greater than 10cm DBH, regardless of whether the tree crown can be seen in the remote sensing image data. While understory tree detection is an important area of future work, the scope of this benchmark is focused on crowns in the canopy that are visible from above. It is important to separate the computer vision tasks from a particular ecological goal, such as tree enumeration, to maximize transparency in evaluation and build towards general models that can be used for a variety of ecological applications. Once algorithm performance is adequate for canopy crowns, additional sources of information will be needed to estimate understory density [20].

We cleaned the raw stem plot data and filtered the data set to contain only stems estimated to be canopy crowns using field-measured height, NEON’s crown position field measurement of sun exposure, and visual interpretation. A stem had to meet the following criteria: 1) had valid spatial coordinates, 2) had a unique height measurement within each sampling period (some trees were recorded twice with different heights and were discarded), 3) was sampled in more than one year and have height changes between years of less than 6m, 4) was classified as alive, 5) when a NEON field record did have a recorded canopy position, that position was not ‘shaded’, 6) had a minimum height of 3m (to match the threshold in the remote sensing workflow), and 7) was no more than 5m shorter than the canopy height model extracted at the stem location to prevent matching including understory trees.

Methods that perform optimally on the field-collected stems evaluation data will predict a single bounding box that contains a single field-collected stem. For each field plot we score the proportion of field stems that fall within a single predicted crown. Field stems can only be assigned to one crown prediction, such that if two crown predictions overlap a single field stem, only one crown prediction is considered a positive match. The resulting proportion of stems with a positive match can be used to estimate the stem recall rate, ranging from 0 (no correctly matched stems) to 1 (all stems are matched).

### Training annotations

During our research on canopy crown detection algorithms [11, 35], we annotated many geographic tiles separate from the evaluation data [36]. The training sites were selected to capture a range of forest conditions including oak woodland (NEON site: SJER), mixed pine (TEAK), alpine forest (NIWO), riparian woodlands (LENO), southern pinelands (OSBS), and eastern deciduous forest (MLBS). The training tiles were chosen at random from the NEON data portal, with the requirement that they did not contain a large amount of missing data and they did not overlap with any evaluation plots. Depending on the tree density at the site, we either annotated the entire 1 km^2^ tile or cropped it to a smaller size to create more tractable sizes for annotation. This data is released alongside the benchmark dataset; however, our goal is to promote the best possible crown-delineation algorithm regardless of training data, and it is not necessary to use this training data to generate predictions. Given the large size of training tiles, the training annotations were less thoroughly reviewed and were only based on the RGB imagery.

### Uncertainty in annotations

#### Differences between image-only annotators

**Fig 7:**
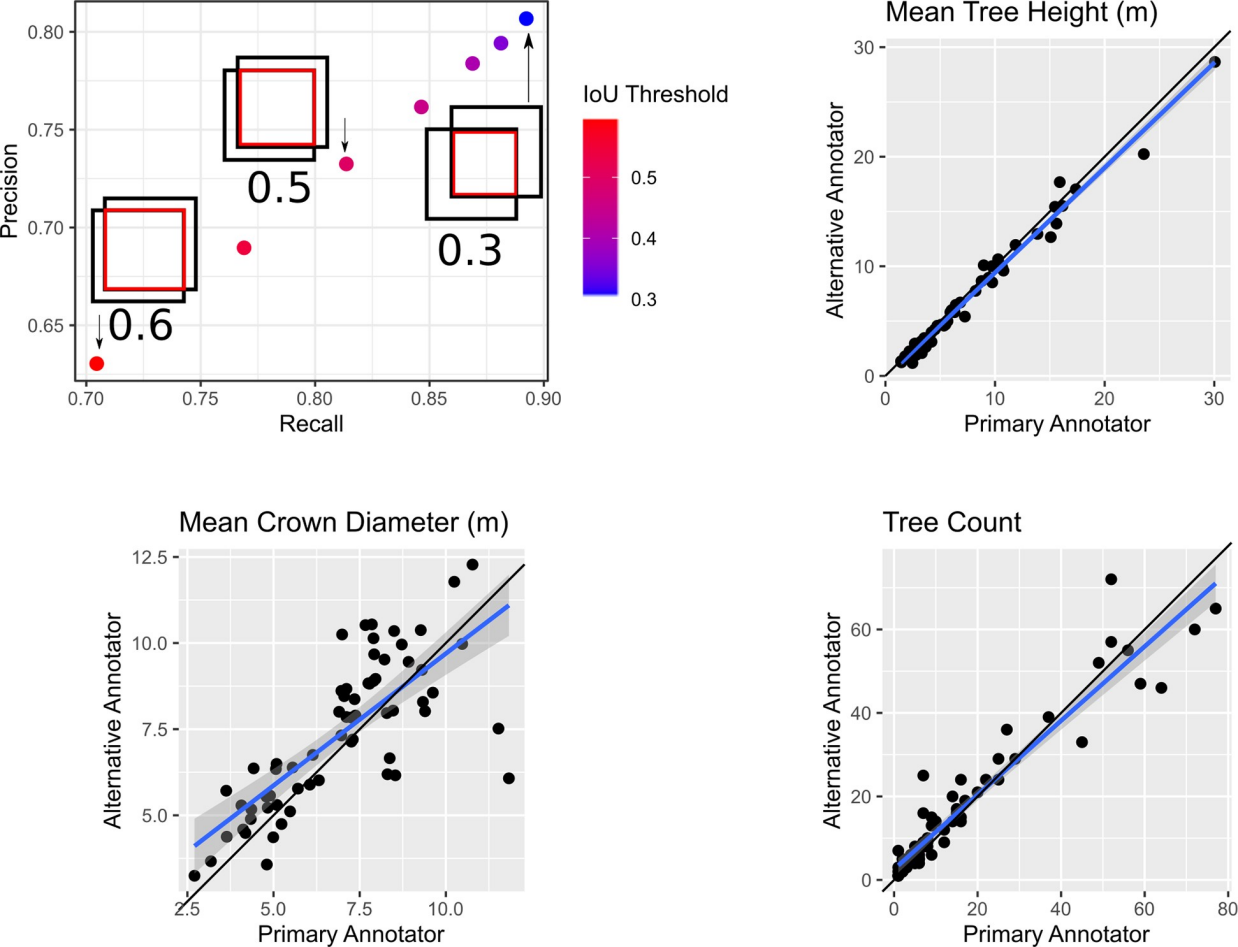
Intersection-over-union scores (top left), as well as plot-level inferences, between the primary annotator and a 2nd annotator. For the IoU scores, we plotted precision and recall for 7 different intersection-over-union thresholds. As the overlap threshold decreases, the two annotators tend to agree on ground truth tree crowns. Analysis is based on 71 evaluation images (n = 1172 trees) that were separately annotated by two different annotators.

Since the image-annotated crowns were done by visually inspecting the images, the exact position and number of bounding boxes in an image depends on the annotators’ interpretation of the image and identification of crowns. Image interpretation is a standard practice for creating validation sets in remote sensing (e.g.[37]), but depends on the skill of the annotator and always introduces uncertainty to validation [38]. In many computer vision tasks, class boundaries are clear and definitive. However, the combination of image quality, spatially overlapping crowns and the two-dimensional view of a complex three-dimensional canopy makes it difficult to identify where one crown ends and another begins. To assess this uncertainty between image annotators, a second annotator annotated 71 evaluation plots using the same data as the primary annotator. We then compared these annotations using a range of intersection-over-union (IoU) thresholds to indicate crowns that matched between annotators (Fig 7). We found that crown matches (recall) among annotators ranged from approximately 70% at lower IoU thresholds to 90% at higher IoU thresholds. This variance indicates that differences between annotators reflect differences in crown extent, rather than differences in whether or not a tree is present. If tree detection was the primary area of disagreement changing the IoU threshold would have minimal effect on the recall and precision rates. This was also supported at the plot level, where the number of trees and mean tree height determined from the LiDAR cloud were very similar across multiple annotators, but there was more variation in the mean crown area (Fig 7).

#### Comparison among image-annotated and field-annotated crowns

**Fig 8:**
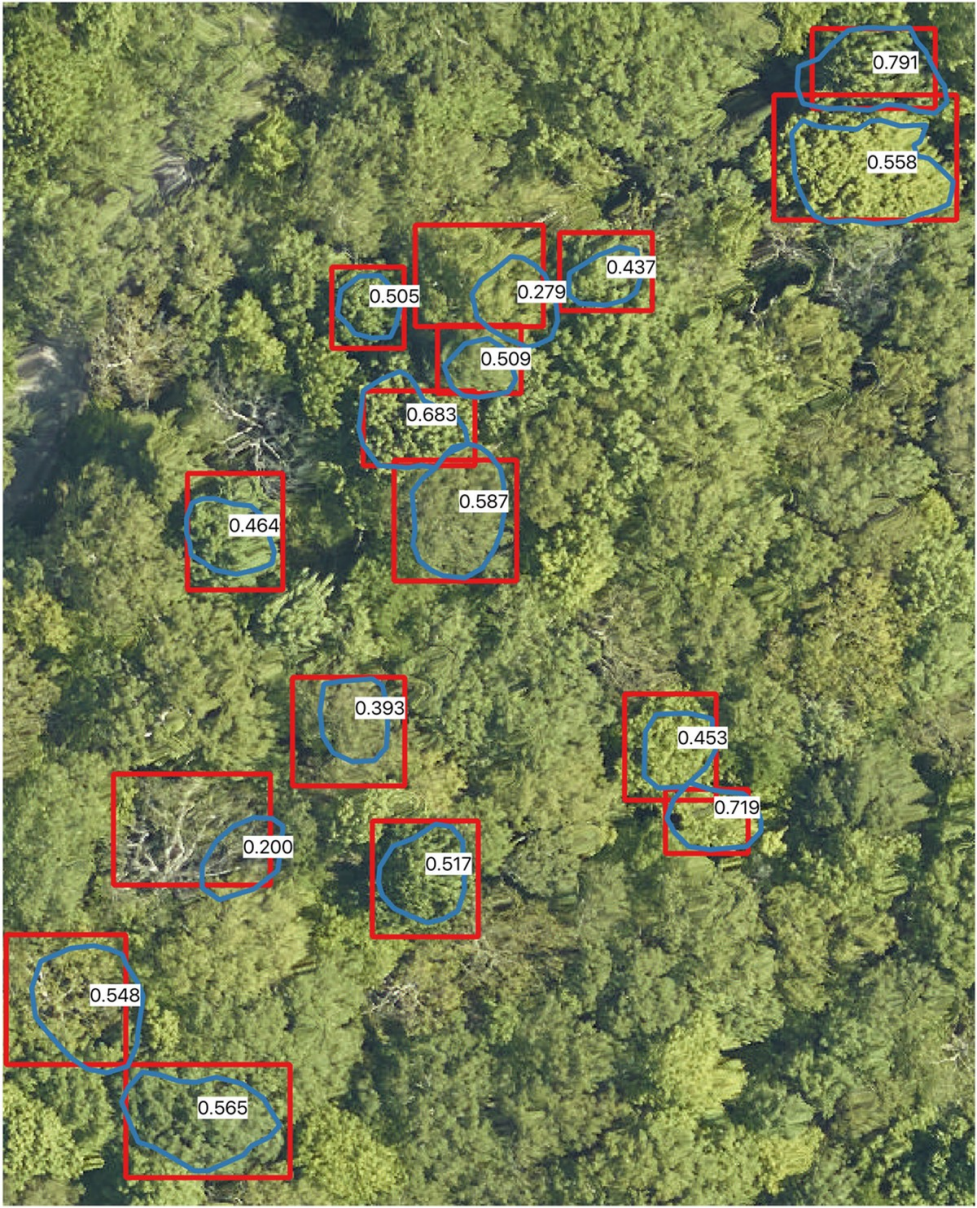
Comparison of field-annotated crowns made by one author (SG) in blue (n = 16) and image-annotated crowns made by another author (BW) in red at Mountain Lake Biological Station, Virginia. Intersection-over-union scores are shown in white. Only the image-annotated crowns associated with the field crowns are shown (out of the 206 image-annotated crowns in this image). From this and similar visualizations we determined that a threshold of 0.4 was a reasonable choice for eliminating crowns that are not sufficiently overlapping to be used for ecological analysis.

To assess the ability for image-annotated crowns to represent field validated data, we compared image-annotation made by the primary annotator (BW) with the field-annotated crowns (SG) at two sites for which there was overlapping remote sensing imagery (Fig 8). We compared image annotations and field crowns using the crown recall rate, defined as the proportion of field-annotated crowns that overlap an image-annotated crown (IoU threshold > 0.4), and the stem recall rate, defined as the proportion of field-annotated crown centroids that are within a single image-annotated bounding box. The primary annotator independently annotated 1553 crowns in images that overlapped with 91 field collected crowns at Mountain Lake Biological Station (MLBS) and 27 crowns at Ordway-Swisher Biological Station (OSBS). To prevent the annotator identifying the obvious location of the field crown, the test image encompassed a large area. Using field-annotated crowns as ground truth, the image annotations had a stem recall rate of 96.7% indicating that image annotation can identify the presence of trees in all but rare cases. There was more disagreement in the extent of crown boundaries. The image-annotated crowns had a crown recall of 78.0% with the field-annotated crown polygons. While we anticipated greater accuracy for large field-annotated crowns, we found only a modest relationship between crown area of field-annotated crowns and correct image-annotated match. In general, errors tend to be marginally biased towards oversegmentation, where large crowns are divided into smaller sets of branches, but both types of errors occur in relatively similar frequencies.

### NeonTreeEvaluation R Package

To maximize the value of the benchmark dataset and standardize evaluation procedures, we developed an R package (https://github.com/weecology/NeonTreeEvaluation_package) for downloading the evaluation data and running the evaluation workflows. This package takes a standard submission format of predicted crowns in either bounding box or polygons as input and returns the evaluation scores of the detections for each of the three evaluation datasets. This reproducible workflow will facilitate creating a transparent process for future comparisons among crown detection algorithms.

**Fig 9:**
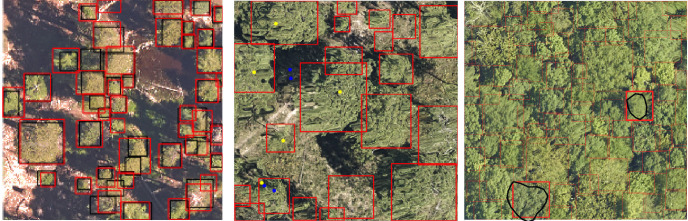
Example predictions using the DeepForest algorithm. Left) DeepForest predictions in red and compared to image-annotated crowns in black from Teakettle Canyon, California. Middle) DeepForest predictions in red are compared to field-collected stems, with matching stems in yellow and missing stems in blue, from Jones Ecological Research Center, Georgia. Right) DeepForest predictions in red with the field-annotated crown in black from Mountain Lake Biological Station, Virginia. The matching prediction is shown in bold while the other predictions are faded for visibility.

To demonstrate the performance of a detection method on the benchmark dataset and allow for users to gauge their performance against published methods, we used the DeepForest Python package to generate crown detections in the benchmark sensor data [34]. DeepForest is a RGB deep learning model that predicts canopy crown bounding boxes [11, 23, 35]. The prebuilt model in DeepForest was trained with the training data described above, but did not use or overlap spatially with any evaluation data in this benchmark. Following the best practices for computational biology benchmarking described in [13], we emphasize that the DeepForest algorithm was designed in conjunction with these evaluation data and it is therefore not surprising that it performs well, with image-annotated boxes and field-annotated crown polygons both at approximately 70% accuracy (Table 3, Fig 9). It is also notable that despite the uncertainty with the crown area of the image-annotated crowns, the overall score is similar among evaluation data types.

**Table 3:** Benchmark evaluation scores for the DeepForest python package.

Image-annotated Crowns		Field-annotated Crowns	Field-collected Stems
Recall	Precision	Recall	Recall
79.0	65.9	72.2	74.0

## Discussion

This benchmark provides annotations, data and evaluation procedures for canopy crown detection using multiple sensor types across a broad range of forest ecosystems. The inclusion of multiple evaluation types is critical because each type of evaluation data has strengths and limitations in evaluating model performance. Field collected stems are the most common evaluation data used in crown detection work due to high confidence that each stem represents a location of a single tree [1, 6, 17, 39]. However, the position of a tree stem can fail to accurately represent the position of the crown as viewed from above due to a combination of spatial errors in alignment with the image data and the tendency for trees to grow at acute angles (tree lean is not measured in the NEON data), such that the center of the crown and position of the stem can be offset by several meters. A second limitation of field-collected stem point locations as evaluation data is that they are typically collected for only a portion of the trees in the landscape covered by a remote sensing image. This makes it difficult to calculate model precision, since it is not possible to differentiate a non-matching prediction of a tree crown from a correct prediction of a tree crown that lacks stem data. Therefore, evaluating tree crown algorithms without evaluating precision has the potential to reward algorithms that include many spurious crowns. In contrast, image-annotated crowns are relatively easy to scale, allowing the collection of data for a wide range of forest types and for annotation of every visible crown in the image. Using image-annotated crowns supports the evaluation of methods across a broad range of forest types and allows both recall and precision to be calculated. However, since these annotations are not generated by an observer in the field there can be errors due to interpreting the images [32]. This problem is solved using field-annotated crowns in which an observer annotates the remote-sensing imagery on a tablet while in the field [33]. The main limitation to this approach is that it is labor intensive, meaning that only a relatively small amount of validation data can be collected, making it difficult to obtain a large number of crowns across broad scales or assess model precision. Given the tradeoffs in each evaluation type, providing multiple criteria is a useful way of balancing the need for broad scale model verification with rigorous evaluation of field-based measurements.

While they are often analyzed separately, this benchmark dataset includes aligned data from RGB, LiDAR and hyperspectral sensors for a range of geographic areas because each of these data types may be useful for canopy crown detection. Three-dimensional LiDAR data has high spatial resolution, but lack of spectral information makes it difficult to identify tree boundaries. RGB data has spectral information and high spatial resolution but lacks context on vertical shape and height. Hyperspectral data is useful for differentiating individual crowns based on differences in foliar properties driving by differences in tree species or structure, but generally has a coarser spatial resolution. Combining sensor data may lead to more robust and generalizable models of tree detection at broad scales, which makes having all three data types aligned an important component of a forward-looking benchmark dataset. While the NEON dataset differs from other airborne collected data products in image resolution and details of data acquisition, it offers a large range of forest types and standardization of evaluation metrics. However, the benchmark notably lacks examples from forests outside of the United States, including tropical forests that are of high conservation concern. Researchers interested in generalizing to areas outside of the NEON sites can use this data to first validate algorithms on a known benchmark before applying it to novel landscapes.

This benchmark is focused on the task of canopy tree detection. This is only one step in the broader ecological task of inferring total tree counts or functional characteristics of forests from airborne data. There remain significant hurdles to convert canopy tree crowns into total tree counts that include understory stems, especially across forest types. For example, NEON uses a 10cm DBH cutoff for field stems. This size cutoff corresponds to different ecological roles in different ecosystems and should itself not be seen as a total count. To make this benchmark applicable to a wide variety of applications, we have not included understory ecological measures in the evaluation metrics since none of the sensor data directly detect understory trees, but encourage the development of future benchmarks in this area that are designed to facilitate applications requiring understory information. For example, simulating latent tree size distributions from observed data is a promising avenue to interpolate canopy trees visible in airborne images to full tree size class distributions [25]. Given the current performance of available algorithms, we believe substantial improvement is needed in canopy detection before moving to the more difficult understory detection task.

While the annotations in this dataset are all two dimensional and some are represented only by bounding boxes (the image-annotated crowns), there are opportunities to extend the benchmark dataset into new formats and dimensions. For example, there has been recent interest in object detection using input rasters, both as a replacement for traditional bounding boxes, and as an additional step in refining pixel-based contours of object boundaries [40]. By rasterizing the annotated bounding boxes, the dataset can be used to compare segmentation strategies such as raster-based versus regional proposal networks [41] and matches more directly with polygon-based approaches to annotating crowns. Furthermore, combining 2D optical data and 3D point cloud annotations remains an active area of model development [42]. Trees have complex 3D and 2D representations and the data provided in this benchmark could be used to develop new evaluation procedures across dimensions.

By providing a repeatable evaluation workflow, we hope to reduce the uncertainty in novel algorithm development and promote model and data sharing among researchers. Initial work in [43] showed that deep learning algorithms can learn from multiple geographies simultaneously, without losing accuracy on the local forest type. This means that data sharing among researchers can provide mutual benefit to all applications, even from disparate forest types. By standardizing evaluation criteria, we hope to foster collaboration and comparative studies to improve the accuracy, generalization, and transparency of canopy crown detection.
