# Supplementary material for: A benchmark dataset for canopy crown detection and delineation in co-registered airborne RGB, LiDAR and hyperspectral imagery from the National Ecological Observation Network
Source: PLoS Comput Biol. 2021 Jul 2;17(7):e1009180. doi: 10.1371/journal.pcbi.1009180 (PMC8282040; doi:10.1371/journal.pcbi.1009180)
Supplement: Attachment — Submitted filename: review 220221.docx [file pcbi.1009180.s001.docx]

This manuscript presents an openly available dataset to allow evaluation of tree detection algorithms which work from remotely sensed data. This is a heavily studied problem with key implications for forest management and its role in climate change mitigation. However, to date there have been limited studies and benchmarks and where these have been completed the data often remains closed, of limited data types and replicating the benchmark for new methods is hard. I therefore commend the authors for their effort in compiling and sharing both a dataset which addresses several ways to approach tree detection and an accompanying R package to allow evaluation. This benchmark should form a key part of future protocols of evaluation as the field of remote sensing of tree locations continues to improve, noting there is still no gold-standard of method to detect trees. This is certainly a key dataset and tool which will have great value to many in the fields of remote sensing and forest ecology and I believe will be of great interest as a fully complete pipeline for method validation. Overall, I think this dataset and manuscript accompanying it are of great value to the field, and whilst not perfect, form a key step of progress in efforts to have an agreed benchmark for proposed methodologies. The size and mixture of sites in the dataset should allow comparison of more complex and advanced approaches from different imagery sources and will enable the field to advance and better understand the strengths and weaknesses of each method.

I will now address the comments, questions, and thoughts I have going through the manuscript relating to the accompanying dataset.

Overall, I think this manuscript shows a clear introduction to the dataset, and critically, highlights many of the limitations, which are common for similar benchmarking studies if not as openly stated.

Line 78: For all NEON datasets you explain some of how the data was processed. It was not initially clear to me which processing was complete by NEON and which by the authors. Some changes to wording might help clarify what modification, if any, was part of preparing the dataset.

Line 101: Did the LiDAR data arrive normalised, or did you subtract the ground? Whilst a common step for forest modelling, this can introduce some crown warping on steep terrains which can affect extraction and statistics of crowns using this data. For example, of this discussion see Vega et al 2014. Could be worth briefly touching on this even if the data is all normalised.

Line 121: Figure 3 – could you say which of the RGB channels each band of the hyperspectral imagery was mapped to, it’s not clear in the caption.

Line 152 and line 240: Here there is an in-text citation in authoryear format, make sure you adjust this to numbered notation like the rest of the text.

Line 154: I would argue that recall is not ‘model accuracy’. This is part of model accuracy and you should make this distinction clearer. As an extreme example, marking every pixel in an image as a distinct crown would have terrible accuracy but would have perfect recall.

Line 157: ‘This gap can be critical since an algorithm covering an image in many predictions is likely to have high accuracy’ only if you define accuracy as above. This is an issue which other benchmarking studies have already highlighted in terms of over and under detection/segmentation so maybe you should consider citing them (eg Eysn et al 2015 and Aubry-Kientz et al 2019)

Line 162 -169: You argue that the remotely annotated imagery can be used to evaluate precision and recall. Yet in the later part of this text, you highlight that the accuracy of the manual annotation itself can introduce biases, does this not affect the ability of this dataset to be able to measure both recall and precision, given that any errors in the manual annotation propagate into these measures?

Line 187: ‘Field stems can only be assigned to one prediction, such that if two predictions overlap a single field stem, only one is considered a positive match’. Is this not the same issue as in line 154, whereby a terribly imprecise method is not penalised? Did you consider some metric of number of crowns potentially attachable to each stem as a proxy of precision? Similarly, have you visually inspected the imagery to make sure each stem is identifiable to one crown only? Seems like a step that might be important to ensure there is some link between the imagery and stem inventory.

Line 198: I agree bounding boxes are common in computer vision and so a dataset of this nature could be useful. However, many tree detection algorithms focus on the actual crown extent as you have in your field mapped crowns. The obvious benefit of your approach is speed of annotation (which I would argue increases the risks of bias introduced by errors given the time per crown). Did you consider using convex hulls or fully drawn crowns as a better measure of tree crown delineation (which will have consequences for biomass, growth, and phenology modelling). For example, the benchmark in Marconi et al 2019 was focused on ‘true’ crown shapes (I note that this was a study written by many authors of this work). Arguably this is the dataset that would be most valuable for benchmarking tree crown detection. As detection is only the first part of the story for actual applications to forest ecology and management. What do you think?

Line 221: ‘we annotated all tree regardless of health status’. Why did you change this from the stems dataset where only alive trees were kept? Is this not a source of potential bias between your evaluations?

Line 224: Same as line 198, you argue your approach is in keeping with computer visions but less so crown extraction? Is this not a limitation once we come to assessing methods for application to tracking changes to individual trees (the ultimate goal for most methods)?

Line 249: Rather than a fixed cut-off for IoU for ‘correct’ or ‘wrong’ detections, did you consider returning a histogram or similar of relative frequency of IoU scores? Seems like this is a rich way to compare strengths of methods rather than a semi-arbitrary cut-off for which you haven’t shown a sensitivity analysis (or maybe a precision-recall curve or similar would be good to justify your cut-off)

Line 298-299: Whilst I commend your efforts to include all data, including the ‘rougher’ data from your own work, do you not think only delineating on RGB data in your ‘training’ data might be a source of major bias, given you argue the importance of using all data sources in mapping crowns. Might be worth highlighting this before the deep-learning community start working on the dataset as a ‘true’ base line.

Line 301: Difference between image-only annotators. Can you give a final agreement percentage for the IoU you chose? It is not clear how much your two annotators agreed in the final dataset. Do you not think that agreement in the range of 80% or so is rather worrying, with 1 in 5 trees missing between two annotators? Surely this shows the importance of proper in field validation. I do however think this sensitivity analysis is crucial and a good indication of the quality of your data and you should be clearer on the final agreement you get with your parameters with discussion of the importance of this for propagating biases in assessing methods on your data.

Line 341: Similarly, do you not think 78% agreement is low to be assessing precision with the output of your annotation? Please correct me if I’m getting this wrong but does this not mean 1 in 5 crowns aren’t properly annotated and thus precision and recall could be as wrong as 20% for a theoretical perfect tree detector.

Line 345: I would like to see some form of sensitivity analysis to justify the 0.4 IoU threshold you settled on rather than visual inspection.

Line 373: Am I correct in thinking the only dataset for which you measure precision is the one with no reference to the field inventory? I understand this is because it is the only ‘full’ survey, but the previous discussion of error and biases make me uneasy with the idea that this is the gold standard for precision. I think it important that you are clear in the discussion of reasons for this dataset to be the one with most probability error in evaluation since field-based methods are tied to validated trees.

Line 396: Why do you think the recall is highest for the dataset which does not link to field-measurement. This is interesting to me that the recall is higher with more crowns mapped in the evaluation step. Any idea why this happened?

Line 426: You talk about recent interest in methods without references. It would be helpful to include references to some work looking at this.

Line 509: S1 Why did you not include dead trees? You include these in annotations in other sections of the dataset and these are arguably of great interest for forest ecology.

REFERENCES

Aubry-Kientz, Mélaine et al 2019. "A Comparative Assessment of the Performance of Individual Tree Crowns Delineation Algorithms from ALS Data in Tropical Forests" Remote Sens. 11, no. 9: 1086. <https://doi.org/10.3390/rs11091086>

Eysn, L et al. A benchmark of lidar-based single tree detection methods using heterogeneous forest data from the Alpine Space. Forests 2015,

Marconi S et al. 2019. A data science challenge for converting airborne remote sensing data into ecological information. PeerJ 6:e5843 <https://doi.org/10.7717/peerj.5843>

C. Vega, et al PTrees: A point-based approach to forest tree extraction from lidar data, International Journal of Applied Earth Observation and Geoinformation, Volume 33, 2014, Pages 98-108, https://doi.org/10.1016/j.jag.2014.05.001.
