# Supplementary material for: A benchmark dataset for canopy crown detection and delineation in co-registered airborne RGB, LiDAR and hyperspectral imagery from the National Ecological Observation Network
Source: PLoS Comput Biol. 2021 Jul 2;17(7):e1009180. doi: 10.1371/journal.pcbi.1009180 (PMC8282040; doi:10.1371/journal.pcbi.1009180)
Supplement: Attachment — Submitted filename: Reviews Benchmark PLOS Computational Biology.docx [file pcbi.1009180.s002.docx]

Dear Dr. Weinstein,

Thank you very much for submitting your manuscript "A benchmark dataset for individual tree crown delineation in co-registered airborne RGB, LiDAR and hyperspectral imagery from the National Ecological Observation Network" for consideration at PLOS Computational Biology.

As with all papers reviewed by the journal, your manuscript was reviewed by members of the editorial board and by several independent reviewers. In light of the reviews (below this email), we would like to invite the resubmission of a significantly-revised version that takes into account the reviewers' comments.

We cannot make any decision about publication until we have seen the revised manuscript and your response to the reviewers' comments. Your revised manuscript is also likely to be sent to reviewers for further evaluation.

When you are ready to resubmit, please upload the following:

[1] A letter containing a detailed list of your responses to the review comments and a description of the changes you have made in the manuscript. Please note while forming your response, if your article is accepted, you may have the opportunity to make the peer review history publicly available. The record will include editor decision letters (with reviews) and your responses to reviewer comments. If eligible, we will contact you to opt in or out.

[2] Two versions of the revised manuscript: one with either highlights or tracked changes denoting where the text has been changed; the other a clean version (uploaded as the manuscript file).

Important additional instructions are given below your reviewer comments.

Please prepare and submit your revised manuscript within 60 days. If you anticipate any delay, please let us know the expected resubmission date by replying to this email. Please note that revised manuscripts received after the 60-day due date may require evaluation and peer review similar to newly submitted manuscripts.

Thank you again for your submission. We hope that our editorial process has been constructive so far, and we welcome your feedback at any time. Please don't hesitate to contact us if you have any questions or comments.

Sincerely,

Jacopo Grilli

Associate Editor

PLOS Computational Biology

Bjoern Peters

Benchmarking Editor

PLOS Computational Biology

**Dear Drs Grilli and Peters,**

**Thank you for the opportunity to improve on this manuscript. The recommendations of the reviewers were useful for helping us clarify the goals, approaches, and limitations of this benchmark dataset.**

**Overall we have 1) tightened the language to communicate more clearly by reducing jargon and be more consistent in our terminology, 2) provided overview tables of the evaluation data and NEON sites, 3) added additional text highlighting the strengths and weaknesses and the resulting complementary nature of the multiple evaluation types, 4) expanded the description and improved the citation of the NEON data products, and 5) clarified that, as with many remote sensing approaches, the scope of the benchmark is limited to canopy (i.e., sun-exposed) trees.**

**Please see detailed responses to each reviewer below, as well as the attached track changes document.**

# Reviewer 1

This work introduces a remarkable (and necessary) effort to standardize the evaluation of individual tree segmentation algorithms. I believe that the paper is overall very clear and therefore I only have minor remarks.

Methodological remarks

Main main methodological remark concerns the selection of the evaluation sites: the choice of the evaluation sites could use more quantitative methods (e.g., scene descriptors as suggested by Oliva and Torralba 10.1023/A:1011139631724) than just a vague "to represent a breadth of forest landscapes across the continental US" (line 194). The same holds for the selection of the training tiles (lines 287-293): how are the forest conditions selected? annotations? could these characteristics be computed with scene descriptors?

**The training sites from the seven NEON sites were selected based on the following criteria, 1) there was high quality RGB data that was geographically separate from any overlapping areas of NEON field plots used for validation, 2) they represented as many major biomes covered by NEON as possible. Following the reviewer’s request we have expressed this as a more quantitative metric using tree density per 20m subplot in the NEON sampling plots. We added L172 to explain this range.**

**The validation data included validation plots paired to training plots at the same NEON sites; in addition as many new NEON sites were added that had high quality data in all sensors. We did not include predominantly treeless sites, such as in the desert Southwest and Alaskan tundra.**

This issue is also relevant to the issue of generalization discussed in lines 410-414: having a low-dimensional numerical representation of a scene could serve as a quantifiable basis to assess whether a given algorithm trained based on certain tiles might perform well under unseen scenes (i.e., in this case, forest conditions). I understand that incorporating such a feature into the existing package might be quite complex and I believe that the article can be published without it. Nevertheless, it seems that the package could benefit from scene descriptors in a near future, and I thus encourage the authors to consider how they could be exploited (if they have not considered it already).

**We agree that this would be a valuable addition, but also that it is a contribution for a future update to the benchmark for generalizing to new sites. We are currently prioritizing models with some local data, as this remains a difficult problem in the field** [**[1,2]**](https://www.zotero.org/google-docs/?UoiwV2)**.**

* The abstract and introduction could make explicit that the benchmarks only concerns the (continental?) United States of America (more than just stating "National Ecological Observation Network", since "National" could mean many nations).

**We added “covering dominant forest types in the United States” to the abstract. L25**

* In the introduction, the authors should better develop the sentences in the lines 55-58 and 59: first, the adjective "good" (for A good benchmark..." can have very subjective connotations. Secondly, are the "three components" described in lines 55-58 based on any specific study (maybe the preceding references 12-13)? The authors should make clearer where these criteria come from. The same goes for the "three objectives" alleged in line 59.

**We altered the text to make the opinion explicit. L56 “** We believe a useful benchmark dataset has at least three features [[19–22]](https://www.zotero.org/google-docs/?7DRhaY)**...”**

* If possible, in the discussion, the sentences of lines 425-428 (from " For example," to "contours of object boundaries"), and lines 428-431 (from "By rasterizing" to "area of model development") should add some references to support their respective statements.

**We have added citations and more concrete examples to L395, L397.**

* closing parenthesis mismatch in line 286

**This text was corrected.**

* use capital "P" for Python in line 383

**This text was corrected.**

# Reviewer 2

This manuscript presents an openly available dataset to allow evaluation of tree detection algorithms which work from remotely sensed data. This is a heavily studied problem with key implications for forest management and its role in climate change mitigation. However, to date there have been limited studies and benchmarks and where these have been completed the data often remains closed, of limited data types and replicating the benchmark for new methods is hard. I therefore commend the authors for their effort in compiling and sharing both a dataset which addresses several ways to approach tree detection and an accompanying R package to allow evaluation. This benchmark should form a key part of future protocols of evaluation as the field of remote sensing of tree locations continues to improve, noting there is still no gold-standard of method to detect trees. This is certainly a key dataset and tool which will have great value to many in the fields of remote sensing and forest ecology and I believe will be of great interest as a fully complete pipeline for method validation. Overall, I think this dataset and manuscript accompanying it are of great value to the field, and whilst not perfect, form a key step of progress in efforts to have an agreed benchmark for proposed methodologies. The size and mixture of sites in the dataset should allow comparison of more complex and advanced approaches from different imagery sources and will enable the field to advance and better understand the strengths and weaknesses of each method.

**We thank the reviewer for the feedback.**

# Reviewer #3

A benchmark dataset for individual tree crown delineation in co-registered airborne RGB, LiDAR and hyperspectral imagery from the National Ecological Observation Network

The authors describe a set of datasets that would be useful for assessing accuracy of tree segmentation from either aerial image data only or from mixed sets of data (RGB, lidar, hyperspectral). The data provided appears to be specific to the NEON research sites, although it may also be useful for a limited number of other research-grade aerial sets of data. The work appears to assume a specific workflow from aerial imagery.

This is a useful dataset, but I recommend that the authors revise the manuscript to provide more connections between the individual data products and the context of the workflow assumed by these datasets. For example, how many of the image tree segmentation methods (the authors state that there are many) would use this range of data products?

**The goal of this dataset is to make as many types of data available to foster innovation in crown detection while promoting reproducible evaluation scores. Each data product provided is used in published algorithms demonstrating their relevance for tree crown detection. By providing all of these data products together this allows algorithms based on any underlying data product, or combination of data products, to be directly compared. We do not assume any particular workflow, only that the final prediction be in the form of a tree crown box or polygon. We apologize if this was unclear and have added text early in the introduction to clarify this (L67).**

The descriptions of each of the data products is sparse. This may be in keeping with the style of the journal. However, for a reader who is not familiar with the segmentation workflow that would use each of these products, I suspect that they would have to puzzle over the descriptions to fully understand the data products.

**We have added additional detail on each data product and evaluation annotation and updated the references to each of the NEON documents. While we agree that expansion was useful, the full description of all of the relevant NEON products, provided by NEON, represents hundreds of pages of documentation and therefore is better accessed via the citations provided in the text.**

I approached this manuscript from the perspective of a researcher who has spent many years segmenting trees from airborne lidar data. I had assumed that this dataset was a generalized dataset that could be used for training and testing of segmentation methods that included both lidar and image data. It became clear that the dataset, and the metrics provided, are heavily oriented toward image tree segmentation. The authors should make this clear both in the abstract and the beginning of the introduction.

**Since the largest source of ground truth is ‘image-annotated crowns’, we use images as the central dataset, since it reduces the potential for cross sensor georectification error. NEON’s LiDAR data is only 5pts/meter and often less than that. It is a valuable source of information, but is somewhat different to the hundreds of points per meter featured in many recent LiDAR segmentation publications** [**[e.g., 7]**](https://www.zotero.org/google-docs/?rSmQyW)**. We have highlighted this on L112. Nevertheless, we believe an optimal solution will require data integration among all sensor types. This benchmark prioritizes geographic variation in forest conditions, rather than evaluation data in all sensor types. We added L66 explaining our perspective, as well as added a paragraph on the tradeoffs among sensor types in the discussion L359.**

In addition, as the authors point out, there are many methods for segmenting trees from image data. The authors should be clear about the generality of this data to image segmentation methods. The authors appear to have tuned this dataset to a particular image segmentation methodology. They should make this clear and describe that method and provide a flowchart showing how each of the dataset’s products would be used in that workflow.

**We have clarified the text. We have not tuned the workflow to assume any combination of data or sensors (L54). As long as a method produces bounding box or polygon predictions it can be assessed using the evaluation routine. Users can select one or multiple data products that best suit their needs (L62).**

The authors present a number of different products one-by-one. As I read this manuscript, it is hard to keep each of these datasets in mind and how they relate to each other. I recommend that the authors provide an introductory section that describes all the datasets as well as a table or figure that provides a quick reference to the data products. Figure 5 partially addresses this.

**We have added an introductory table to describe the different data components, their formats and extents (Table 1, Table 2).**

The authors should provide a table listing the NEON sites and the data available at each. For example, from Line 268, at least two NEON sites have data not provided for the other sites. Also, the acronyms of the sites should be provided in this table so users can relate these to the site acronyms used throughout the manuscript.

**We have added to Table 2 for the count of each annotation for each site and additional notes on the NEON siteID and location.**

In many ways, this data set is unusual. Relatively few image data sets are rectified to the top of canopy instead of ground. This greatly limits layover effects and hence improves segmentation accuracy. Also, the images used in this dataset have just three color bands, while many image datasets will have four (for example, NAIP imagery). The NEON images are also higher resolution than many regional image sets (such as NAIP or many other regional-to-state/nation scale image sets) The authors should make clear how their image dataset is similar and different from many commonly available datasets. I suspect that these datasets have relatively limited usefulness outside of relatively few research datasets (which the NEON data are).

**As stated above and described in the paper, the RGB image data is only one component of this benchmark dataset. In addition, the fact that other datasets exist that are not identical in spatial resolution or band numbers in no way decreases the need for benchmark datasets. In contrast, it underlines the need for standardization to allow rigorous comparisons among tree detection models. No dataset can perfectly represent the breadth of applications currently in the field, but NEON is the largest airborne data collector for ecological applications that brings together multiple sensors and field validation. The NEON sites cover over 100 million trees spanning the dominant ecosystems in the United States and the network has been specifically designed to support broad cross-scale ecological research. These data can act as a bridge to individual applications by providing validation on a known dataset. We have added text to the discussion to highlight this utility, as well as the need for extension to other important areas and datasets (L372).**

Line 76: The description of the RGB camera characteristics should include the peak wavelength frequencies for each of the spectral bands.

**The details of data collection are covered by the NEON technical documents. The documents are linked in the manuscript.**

Line 91: Could the authors be more specific by what they mean by the center portion of images? Center 25%? 50% 90%

The authors should justify not including portions of images that include greater distortion since these conditions will be encountered by research teams working with these data.

**We did keep portions of images with moderate distortions, “We did not include images with large enough distortions to interfere with tree crown detection but kept images with minor distortions to represent the kind of challenging conditions present in applied settings.”**

**We removed images that were completely unrecognizable, which happens on occasions near the edge of the flightline. They are sufficiently distorted that they could not be used for making predictions L107.**

Line 97: Our experience in using the NEON lidar data sets is that they can have varying quality within a single acquisition. This is causing us problems in using the lidar data for one of the lidar sets included in the authors dataset. I suggest that the authors provide a data layer that records pulse density per square meter to help users assess variations within the lidar data for the supplied datasets.

**We have experienced similar challenges with the widely varying pulse density of the LiDAR across both sites and flightlines. The information that is available on this variation is only present in the point cloud itself (NEON does not provide a separate raster product for pulse density) and therefore this data is already present in the benchmark and accessible to researchers with sufficient experience with LiDAR data to be interested in it. We have added text highlighting the variation in LiDAR densities at broad scales compared to the coverage commonly used at fine scales L118.**

Line 137: Are species also recorded? Also, are snags included in the vegetation dataset?

**Species are not yet recorded in this dataset, but this is certainly important and we have begun work on the species classification aspect that we will release when it is ready.**

**L194: “We annotated all trees, regardless of health status, provided they were standing at the time of data collection. “**

Line 165: The points in this sentence are discussed at length in Jeronimo et al. 2018:

Jeronimo, S., Kane, V.R., McGaughey, R.J., Churchill, D.J., Franklin, J.F. 2018. Characteristics of lidar individual tree detection across structurally diverse forest landscapes: A framework for use in forest management. Journal of Forestry. 116:336-346.

**Added citation to L159.**

Lines 181-184: I am not sure what the authors are trying to say in these sentences.

**We have revised this section to clarify the relationship between NEON field stems and the trees selected as overstory crowns to be used in the evaluation dataset. We have expanded discussion of this on L233 and in the discussion L375.**

Line 185: What method of remote sensing tree identification is being used?

**Line 185, which has now been deleted, read“Using the overstory stem data, the goal is to predict a single bounding box that matches to a single field-validated stem.” We are unsure what the reviewer meant by this comment.**

Line 191: I am finding it hard to follow all the data that is available. I suggest that the author augment Figure 4 with a listing of all the data types, with the values recorded, for an example bounding box and its enclosed tree. Also, do the data include the field-collected stems?

**We have added a summary table at the beginning of the manuscript. Table 1.**

Line 213: What is the purpose of the decorrelation stretch? I see it’s purpose, but the authors might include a sentence, “Decorrelation stretches are useful for…”

**We added an explanation of its utility on L187.**

Line 240: The authors need to describe what is meant by, “area of the overlap”. I suspect that this refers to a specific (image based?) tree segmentation method; it is not something I’ve seen in reading a number of papers on tree segmentation from lidar data.

**We have reorganized the evaluation section to more clearly define terms earlier in the manuscript, both in terms of the terminology used L154 and evaluation procedure L210**

Line 267: How are Field Annotated Crowns different than Field Collected Stems (Line 180)?

**As described in the Field-annotated Crowns section of the manuscript, field annotated crowns are polygons that are drawn onto remote sensing imagery to encompass the pixels associated with the individual tree while a researcher is in the field observing the tree crown. Field collected stems are x,y points marked (using survey methods) by NEON field crews that correspond to the position of the trunk of the tree. We have reorganized this section with clearer headings and overviews to walk the reader more clearly through the datatypes starting on L154.**

Line 378: The authors should provide a flowchart of this workflow.

**We do not understand what is being requested in this comment. Conducting the evaluation only requires running a single function,** evaluate_image_crowns**, as documented in the repository. In other words, given a set of bounding box predictions, there is only one step required. The function handles the entire evaluation routine without need for the user to perform any other steps. The general workflow is described in the github repository (**[**https://github.com/weecology/NeonTreeEvaluation_package**](https://github.com/weecology/NeonTreeEvaluation_package)**).**

# Reviewer #4

In this manuscript the authors present a benchmark dataset and associated R package that can be used to evaluate remote sensing-based tree identification algorithms. The dataset seems very useful and looks to be put together well.

There is a considerable amount of work to do for this to be publishable. First, there are a few technical issues with the methods used. These should either be changed or more thoroughly discussed. Specific suggestions are made below. Second, the writing is disorganized and hard to follow. The manuscript needs to be revised for flow and clarity. Again, specific suggestions are made below. The text frequently uses terms before defining them, or without any definition. It reads as though the authors expect the audience to already have a working understanding of NEON, this project, and all of the associated datasets. In revising, the authors should strive to arrange the text in a chronological order, define every term at its first use, and keep in mind an audience that is totally unfamiliar with this project and its source data.

**We thank the reviewer for pointing out issues with the readability of the manuscript. To address this we have added two tables to the beginning of the manuscript to provide an overview of each data product, give it a consistent name, link it to additional resources, and provide information on the number of annotations available at each NEON site, along with the state and NEON abbreviation. We have improved the consistency of terminology and defined terms and abbreviations before using them. We have expanded the description of NEON and the associated data products to make the paper accessible to readers who are not familiar with NEON.**

General comments

Terminology: the following terms are used interchangeably throughout this manuscript:

Tree crown detection

Individual tree detection

Individual tree segmentation

Individual tree delineation

Crown delineation

Crown prediction

There are probably other variants that I missed. The authors must select a single term and apply it consistently.

**We appreciate this feedback as it caused us to rethink the way we use the terms. We have now adopted more specific terminology and provided a justification to the reader about when to use ‘detection’ versus ‘delineation’. This was helpful in differentiating the datatype as well. Added text to L63, L155, changed the title to include both detection and delineation.**

I appreciate the separate training and testing data. This makes it easy for folks who use this package to do a robust job evaluating their work.

**Thanks!**

This study only covers the United States. It needs to be clearly discussed how this limits the applicability and what it would take to extend this dataset to cover global forests.

**We have added text to the discussion (L372) highlighting the lack of tropical data in the benchmark and the need to extend to global forests, especially once species data are available. While this data set is not global in geographic coverage, the dominant ecosystems with trees in the United States that are included in the benchmark data do share many characteristics with forests around the world.**

Line comments

45-46 strange sentence. Try to express this as a statement instead.

**The sentence was rephrased and split into two L50.**

50-51 too broad a statement, definitely possible within a certain universe/project

**The sentence was removed.**

54 in -> of

**The sentence was removed.**

63 “training and tests splits” doesn’t make sense

**Revised to “test” split.**

59-66 any citation or reasoning for these three objectives? They are good, but not immediately clear why this is the authoritative list

**We have citations to highlight recent articles on benchmarking and recent benchmarks for computer vision tasks for ecology L57.**

76, 97, 108, 125, 132 Neon ID’s should not be in titles. It isn’t explained what they mean. Explain it and put it in a more appropriate location, such as a table

**We have added summary table 1 to give an overview and removed NEON ID’s from the titles.**

83, 94, 104, 118, 129 give proper citation to neon docs instead of inline document names

**We have added citations to technical documents and NEON data products.**

Figure 1 include two or three panels with varying conditions, such as open-mid-dense, or young-mature-old. That way we can understand more about how the imagery captures things

**We have added an additional example to figure 1 and figure 2.**

99, 111 be clearer: 1000 m x 1000 m tiles instead of 1km^2

**The text was corrected.**

Figure 2 same as Figure 1, include two or three panels with contrasting conditions.

**We have added an additional example to figure 1 and figure 2.**

112 Why does it matter that the naming conventions align? What matters is whether the data themselves align. If that is the case, say so.

**This text was removed.**

115 “simultaneously as” does that mean “at the same time as”? Doesn’t sound right. When you edit this, please clarify whether it was actually concurrent collection or just within the same season/year

**We have clarified the sentence. RGB and Hyperspectral data were collected concurrently during the same flight.**

116, 202, 208, 209, 231, 233, 288-290 maybe elsewhere - haven’t introduced sites yet, I don’t have any context for these site name codes

**Added Table 2 to introduce site names and data annotation counts.**

134-136 does “distributed” vs “tower” plot type matter? They both end up being 20x20m plots, just in one case it is split between two subplots. 20x20m is 20x20m, I suggest that this may be extraneous information. If it isn’t be clearer as to why the different types matter.

**This text is useful for those more familiar with NEON site sampling and connects other sources of NEON data. We added a parenthetical statement to explain this is useful information for NEON users but not necessary to understand for use of the benchmark.**

138 you only give units for one of the measurements in this list of measurements. Give it for all or none. I suggest none, it is not important here.

**This text was removed.**

159-169 you allude to it but the major shortcoming of hand delineation is that the delineator is working from the same dataset that the tree id algorithm is working from. Thus, it is not an independent truth dataset. You are actually comparing two ways of interpreting the same data. This can still be useful, but the comparison is fundamentally different than a comparison with field data. With field data you ask, how well does this compare to reality? With hand delineation from imagery you ask, how good of a job does the algorithm do given the information available in the images? Please flesh out a discussion of this distinction.

**We have added an introductory paragraph on the evaluation data to provide an overview of the combination of methods used (L61, L157). The virtue of the image-annotation is the broad geographic scope and clear match to the sensor data. As the reviewer has noted elsewhere, the field stem data is challenging because of the lack of connection between the stem points and the sensor pixels, since trees can grow at acute angles. The best evaluation data is the field-annotated crowns, but these are expensive to collect and therefore generally of limited availability. No one method will be sufficient in mastering tree detection, but each provides an important avenue, which is why we provided three different evaluation datasets. We have added this test to the discussion L359.**

181-182 it is very important not to do this. It is good to separate an analysis based on trees you thought you could find vs trees you didn’t think you could find, but we can’t look at only the easy trees and then call that accuracy. This is similar to the last comment. You need to very carefully define what your benchmark is able to assess. If you remove the small trees, you have no way to assess accuracy of tree identification. Instead, you are assessing how well you can identify trees that you were pretty sure you should be able to identify. That might be a useful thing to do, but it is fundamentally different. Please include all trees in the dataset and flag the overstory trees so that both analyses can be completed. Please also improve your discussion of this topic.

**To clarify, we filtered the field stem data set to remove stems that were clearly understory trees. Given that the image datasets we are using can only detect canopy trees, leaving stems that are clearly in the understory in the evaluation data set would not be an accurate test of canopy tree detection algorithms. The reviewer is mixing the ecological goals of tree detection from the utility of this benchmark. Tree detection is one step in the process of converting sensor data into ecological data. While we agree there is an important ultimate goal, the accuracy of the algorithms need to improve before it can be feasible. There remains significant hurdles to convert tree crowns into total tree counts, especially across forest types. For example, NEON uses a 10cm DBH cutoff for field stems. This size cutoff corresponds to different ecological roles in different ecosystems, and should not be seen as a total count. To make this benchmark applicable to a wide variety of applications, we have not included a specific ecological measure as an evaluation metric. We have added an explanation to the methods and discussion L236, L375.**

222-223 This explanation of using bounding boxes comes way too late. Need to explain the rationale for your methods right up front. And your explanation is not satisfactory. Polygons are much more useful than bounding boxes. Give more reasoning and discuss the tradeoffs

**We have added additional detail to the evaluation section (L154, L212). We do not agree that polygons are inherently superior, since 1) it can be very difficult to determine the precise edges of tree crowns, and 2) the immense labeling effort for polygons translates to a tradeoff in the number of trees and sites that can be annotated. It is also not obvious the number of vertices that are required to standardize among sites. In truth, the state of tree crown detection algorithms is such that the difference between crown polygons versus boxes is relatively minor compared to getting the right number and location of each tree. Much more work is needed before this level of detail differentiates algorithms.**

198-199 “coordinates relative to top-left corner of image” extraneous information

**This text was removed.**

238-251 this is an example of a writing style throughout this manuscript that is hard to read. You give the answer first (0.4 in this case), and then show your work backwards. It would be much easier to read if you were to put things in chronological order. For example, here you should start with the standard in CV literature, then talk about the tests you did, then talk about the number you chose. This manuscript would really benefit from a thorough revision to address backward exposition like this.

**We have rewritten and reduced this section to be more focused and removed figure 9. We have worked to revise the manuscript more generally to improve readability.**

274-276 again, you shouldn’t leave the understory trees out here. We need to know how algorithms do in an absolute sense

**We disagree with this comment. From an ecological perspective, yes it is critical to understand the utility of airborne tree detection on estimating understory. However the first step is to develop algorithms capable of producing accurate overstory trees. Current algorithm performance is not yet able to meet this bar. Without focusing on the individual steps of algorithm performance, it will not be easy to differentiate the performance of overstory segmentation versus understory segmentation. We have changed the paper tile, added text in the methods (L235) and discussion paragraphs (L375) to explain this difference.**

279-283 it is mentioned several times that precision cannot be calculated as though that is the only shortcoming of this type of sampling. But scattered trees as ground truth has many more shortcomings. When the ground dataset is composed of scattered trees it is impossible to know whether the tree identification algorithm actually got the “right” tree, because you have no knowledge of the “wrong” trees. Just getting two boxes to overlap doesn’t necessarily mean that you actually got the right tree for the right reason. It is much more valuable to have small clusters of trees or full maps of contiguous areas. The shortcomings of this sampling need to be discussed in much more detail.

**We agree and have added additional to the evaluation section highlighting the strengths and weaknesses of each approach L359.**

286 citation in different format than others

**We have fixed the citation format.**

293-294 what is the smaller size or range of smaller sizes? Be specific.

**This text was removed.**

339 what is a centroid rate?

**We have rephrased in order to reduce the number of terms. The stem recall rate is described on L254.**

359-361 you give this rationale in the caption and the paragraphs that refer to it, but you already described how you came up with the 0.4 number earlier and made no mention of this process. It is confusing for it to be described in two separate places without reference to one another. I can’t tell if this is actually the same threshold, or a different threshold that just happened to be the same number.

**We have reformatted the two paragraphs to be shorter and clearer. We removed figure 9 and condensed the sections.**

379 “repo” is not a word

**This error was corrected.**

395-396 this table caption is the first place you actually define recall and precision, despite using these terms throughout the manuscript. Definition should be at the first mention

**We added more explicit detail on intersection-over-union to make it clearer on L215.**

Discussion: there are very few citations, even when discussing ideas and methods that warrant citation. For example on line 429, “regional proposal network” needs a citation

**We have added a citation for this research direction.**

S1 explain the process of matching stem locations to bounding boxes in the main text, not in a supplement.

**We have moved the matching stem locations information into the main text.**

Also, this method is quite weak. Calling something a match because a point is within a bounding box is very generous. If possible, improve this method using additional field-based measurements to validate the match. Also, is tree lean measured in the field and accounted for in mapping tree locations as points? If not, please express this as a shortcoming in the text.

**As the reviewer recognizes, obtaining optimal evaluation data for tree crown detection at large scales is challenging. This is why we provide three separate evaluation datasets, each with their own strengths and weaknesses that have been clearly highlighted in the text. The specific issue raised here was addressed in the manuscript and has been expanded on in L340:

“However, the position of these stems can fail to accurately represent the position of the crown as viewed from above due to a combination of spatial errors in alignment with the image data and the tendency of trees to grow at acute angles, such that the center of crowns and position of stems can be offset by several meters”
We have added a parenthetical statement to this existing discussion to specifically note that tree lean is not collected.**

**“**S2 This is important information that belongs in the main text. It is also never cited, so the text frequently refer to sites that are never defined.

**We have added Table 2 to orient the reader to the location of NEON sites and their abbreviations early in the main text.**

**Works Cited**

[1.	Weinstein BG, Marconi S, Bohlman SA, Zare A, White EP. Cross-site learning in deep learning RGB tree crown detection. Ecol Inform. 2020;56: 101061. doi:10.1016/j.ecoinf.2020.101061](https://www.zotero.org/google-docs/?9a6nWn)

[2.	Jeronimo SMA, Kane VR, Churchill DJ, McGaughey RJ, Franklin JF. Applying LiDAR Individual Tree Detection to Management of Structurally Diverse Forest Landscapes. J For. 2018;116: 336–346. doi:10.1093/jofore/fvy023](https://www.zotero.org/google-docs/?9a6nWn)

[3.	Peters B, Brenner SE, Wang E, Slonim D, Kann MG. Putting benchmarks in their rightful place: The heart of computational biology. PLOS Comput Biol. 2018;14: e1006494. doi:10.1371/journal.pcbi.1006494](https://www.zotero.org/google-docs/?9a6nWn)

[4.	Northcutt CG, Athalye A, Mueller J. Pervasive Label Errors in Test Sets Destabilize Machine Learning Benchmarks. ArXiv210314749 Cs Stat. 2021 [cited 12 Apr 2021]. Available: http://arxiv.org/abs/2103.14749](https://www.zotero.org/google-docs/?9a6nWn)

[5.	Weber LM, Saelens W, Cannoodt R, Soneson C, Hapfelmeier A, Gardner PP, et al. Essential guidelines for computational method benchmarking. Genome Biol. 2019;20: 125. doi:10.1186/s13059-019-1738-8](https://www.zotero.org/google-docs/?9a6nWn)

[6.	Van Horn G, Cole E, Beery S, Wilber K, Belongie S, Mac Aodha O. Benchmarking Representation Learning for Natural World Image Collections. ArXiv210316483 Cs. 2021 [cited 31 Mar 2021]. Available: http://arxiv.org/abs/2103.16483](https://www.zotero.org/google-docs/?9a6nWn)

[7.	Fischer FJ, Labrière N, Vincent G, Hérault B, Alonso A, Memiaghe H, et al. A simulation method to infer tree allometry and forest structure from airborne laser scanning and forest inventories. Remote Sens Environ. 2020;251: 112056. doi:10.1016/j.rse.2020.112056](https://www.zotero.org/google-docs/?9a6nWn)
