# Supplementary material for: A benchmark dataset for canopy crown detection and delineation in co-registered airborne RGB, LiDAR and hyperspectral imagery from the National Ecological Observation Network
Source: PLoS Comput Biol. 2021 Jul 2;17(7):e1009180. doi: 10.1371/journal.pcbi.1009180 (PMC8282040; doi:10.1371/journal.pcbi.1009180)
Supplement: Peer review history

# Peer review: Decision Letter 0

**DOI:** 10.1371/journal.pcbi.1009180.r001

**Authors:** Bjoern Peters, Jacopo Grilli

4 Mar 2021

Dear Dr. Weinstein,

Thank you very much for submitting your manuscript "A benchmark dataset for individual tree crown delineation in co-registered airborne RGB, LiDAR and hyperspectral imagery from the National Ecological Observation Network" for consideration at PLOS Computational Biology.

As with all papers reviewed by the journal, your manuscript was reviewed by members of the editorial board and by several independent reviewers. In light of the reviews (below this email), we would like to invite the resubmission of a significantly-revised version that takes into account the reviewers' comments.

We cannot make any decision about publication until we have seen the revised manuscript and your response to the reviewers' comments. Your revised manuscript is also likely to be sent to reviewers for further evaluation.

When you are ready to resubmit, please upload the following:

[1] A letter containing a detailed list of your responses to the review comments and a description of the changes you have made in the manuscript. Please note while forming your response, if your article is accepted, you may have the opportunity to make the peer review history publicly available. The record will include editor decision letters (with reviews) and your responses to reviewer comments. If eligible, we will contact you to opt in or out.

[2] Two versions of the revised manuscript: one with either highlights or tracked changes denoting where the text has been changed; the other a clean version (uploaded as the manuscript file).

Important additional instructions are given below your reviewer comments.

Please prepare and submit your revised manuscript within 60 days. If you anticipate any delay, please let us know the expected resubmission date by replying to this email. Please note that revised manuscripts received after the 60-day due date may require evaluation and peer review similar to newly submitted manuscripts.

Thank you again for your submission. We hope that our editorial process has been constructive so far, and we welcome your feedback at any time. Please don't hesitate to contact us if you have any questions or comments.

Sincerely,

Jacopo Grilli

Associate Editor

PLOS Computational Biology

Bjoern Peters

Benchmarking Editor

PLOS Computational Biology

***********************

Reviewer's Responses to Questions

**Comments to the Authors:**

**Please note here if the review is uploaded as an attachment.**

Reviewer #1: This work introduces a remarkable (and necessary) effort to standardize the evaluation of individual tree segmentation algorithms. I believe that the paper is overall very clear and therefore I only have minor remarks.

# Methodological remarks

* Main main methodological remark concerns the selection of the evaluation sites: the choice of the evaluation sites could use more quantitative methods (e.g., scene descriptors as suggested by Oliva and Torralba 10.1023/A:1011139631724) than just a vague "to represent a breadth of forest landscapes across the continental US" (line 194). The same holds for the selection of the training tiles (lines 287-293): how are the forest conditions selected? annotations? could these characteristics be computed with scene descriptors? This issue is also relevant to the issue of generalization discussed in lines 410-414: having a low-dimensional numerical representation of a scene could serve as a quantifiable basis to assess whether a given algorithm trained based on certain tiles might perform well under unseen scenes (i.e., in this case, forest conditions). I understand that incorporating such a feature into the existing package might be quite complex and I believe that the article can be published without it. Nevertheless, it seems that the package could benefit from scene descriptors in a near future, and I thus encourage the authors to consider how they could be exploited (if they have not considered it already).

# Other remarks

* The abstract and introduction could make explicit that the benchmarks only concerns the (continental?) United States of America (more than just stating "National Ecological Observation Network", since "National" could mean many nations).

* In the introduction, the authors should better develop the sentences in the lines 55-58 and 59: first, the adjective "good" (for A good benchmark..." can have very subjective connotations. Secondly, are the "three components" described in lines 55-58 based on any specific study (maybe the preceding references 12-13)? The authors should make clearer where these criteria come from. The same goes for the "three objectives" alleged in line 59.

* If possible, in the discussion, the sentences of lines 425-428 (from " For example," to "contours of object boundaries"), and lines 428-431 (from "By rasterizing" to "area of model development") should add some references to support their respective statements.

# Typos

* closing parenthesis mismatch in line 286

* use capital "P" for Python in line 383

Reviewer #2: See the attached for my review

Reviewer #3: PCOMPBIOL-D-20-01875

A benchmark dataset for individual tree crown delineation in co-registered airborne RGB, LiDAR and hyperspectral imagery from the National Ecological Observation Network

The authors describe a set of datasets that would be useful for assessing accuracy of tree segmentation from either aerial image data only or from mixed sets of data (RGB, lidar, hyperspectral). The data provided appears to be specific to the NEON research sites, although it may also be useful for a limited number of other research-grade aerial sets of data. The work appears to assume a specific workflow from aerial imagery.

This is a useful dataset, but I recommend that the authors revise the manuscript to provide more connections between the individual data products and the context of the workflow assumed by these datasets. For example, how many of the image tree segmentation methods (the authors state that there are many) would use this range of data products?

The descriptions of each of the data products is sparse. This may be in keeping with the style of the journal. However, for a reader who is not familiar with the segmentation workflow that would use each of these products, I suspect that they would have to puzzle over the descriptions to fully understand the data products.

I approached this manuscript from the perspective of a researcher who has spent many years segmenting trees from airborne lidar data. I had assumed that this dataset was a generalized dataset that could be used for training and testing of segmentation methods that included both lidar and image data. It became clear that the dataset, and the metrics provided, are heavily oriented toward image tree segmentation. The authors should make this clear both in the abstract and the beginning of the introduction.

In addition, as the authors point out, there are many methods for segmenting trees from image data. The authors should be clear about the generality of this data to image segmentation methods. The authors appear to have tuned this dataset to a particular image segmentation methodology. They should make this clear and describe that method and provide a flowchart showing how each of the dataset’s products would be used in that workflow.

The authors present a number of different products one-by-one. As I read this manuscript, it is hard to keep each of these datasets in mind and how they relate to each other. I recommend that the authors provide an introductory section that describes all the datasets as well as a table or figure that provides a quick reference to the data products. Figure 5 partially addresses this.

The authors should provide a table listing the NEON sites and the data available at each. For example, from Line 268, at least two NEON sites have data not provided for the other sites. Also, the acronyms of the sites should be provided in this table so users can relate these to the site acronyms used throughout the manuscript.

In many ways, this data set is unusual. Relatively few image data sets are rectified to the top of canopy instead of ground. This greatly limits layover effects and hence improves segmentation accuracy. Also, the images used in this dataset have just three color bands, while many image datasets will have four (for example, NAIP imagery). The NEON images are also higher resolution than many regional image sets (such as NAIP or many other regional-to-state/nation scale image sets) The authors should make clear how their image dataset is similar and different from many commonly available datasets. I suspect that these datasets have relatively limited usefulness outside of relatively few research datasets (which the NEON data are).

Line 76: The description of the RGB camera characteristics should include the peak wavelength frequencies for each of the spectral bands.

Line 91: Could the authors be more specific by what they mean by the center portion of images? Center 25%? 50% 90%

The authors should justify not including portions of images that include greater distortion since these conditions will be encountered by research teams working with these data.

Line 97: Our experience in using the NEON lidar data sets is that they can have varying quality within a single acquisition. This is causing us problems in using the lidar data for one of the lidar sets included in the authors dataset. I suggest that the authors provide a data layer that records pulse density per square meter to help users assess variations within the lidar data for the supplied datasets.

Line 137: Are species also recorded? Also, are snags included in the vegetation dataset?

Line 165: The points in this sentence are discussed at length in Jeronimo et al. 2018:

Jeronimo, S., Kane, V.R., McGaughey, R.J., Churchill, D.J., Franklin, J.F. 2018. Characteristics of lidar individual tree detection across structurally diverse forest landscapes: A framework for use in forest management. Journal of Forestry. 116:336-346.

Lines 181-184: I am not sure what the authors are trying to say in these sentences.

Line 185: What method of remote sensing tree identification is being used?

Line 191: I am finding it hard to follow all the data that is available. I suggest that the author augment Figure 4 with a listing of all the data types, with the values recorded, for an example bounding box and its enclosed tree. Also, do the data include the field-collected stems?

Line 213: What is the purpose of the decorrelation stretch? I see it’s purpose, but the authors might include a sentence, “Decorrelation stretches are useful for…”

Line 240: The authors need to describe what is meant by, “area of the overlap”. I suspect that this refers to a specific (image based?) tree segmentation method; it is not something I’ve seen in reading a number of papers on tree segmentation from lidar data.

Line 267: How are Field Annotated Crowns different than Field Collected Stems (Line 180)?

Line 378: The authors should provide a flowchart of this workflow.

Reviewer #4: Overview

In this manuscript the authors present a benchmark dataset and associated R package that can be used to evaluate remote sensing-based tree identification algorithms. The dataset seems very useful and looks to be put together well.

There is a considerable amount of work to do for this to be publishable. First, there are a few technical issues with the methods used. These should either be changed or more thoroughly discussed. Specific suggestions are made below. Second, the writing is disorganized and hard to follow. The manuscript needs to be revised for flow and clarity. Again, specific suggestions are made below. The text frequently uses terms before defining them, or without any definition. It reads as though the authors expect the audience to already have a working understanding of NEON, this project, and all of the associated datasets. In revising, the authors should strive to arrange the text in a chronological order, define every term at its first use, and keep in mind an audience that is totally unfamiliar with this project and its source data.

General comments

Terminology: the following terms are used interchangeably throughout this manuscript:

Tree crown detection

Individual tree detection

Individual tree segmentation

Individual tree delineation

Crown delineation

Crown prediction

There are probably other variants that I missed. The authors must select a single term and apply it consistently.

I appreciate the separate training and testing data. This makes it easy for folks who use this package to do a robust job evaluating their work.

This study only covers the United States. It needs to be clearly discussed how this limits the applicability and what it would take to extend this dataset to cover global forests.

Line comments

45-46 strange sentence. Try to express this as a statement instead.

50-51 too broad a statement, definitely possible within a certain universe/project

54 in -> of

63 “training and tests splits” doesn’t make sense

59-66 any citation or reasoning for these three objectives? They are good, but not immediately clear why this is the authoritative list

76, 97, 108, 125, 132 Neon ID’s should not be in titles. It isn’t explained what they mean. Explain it and put it in a more appropriate location, such as a table

83, 94, 104, 118, 129 give proper citation to neon docs instead of inline document names

Figure 1 include two or three panels with varying conditions, such as open-mid-dense, or young-mature-old. That way we can understand more about how the imagery captures things

99, 111 be clearer: 1000 m x 1000 m tiles instead of 1km^2

Figure 2 same as Figure 1, include two or three panels with contrasting conditions

112 Why does it matter that the naming conventions align? What matters is whether the data themselves align. If that is the case, say so

115 “simultaneously as” does that mean “at the same time as”? Doesn’t sound right. When you edit this, please clarify whether it was actually concurrent collection or just within the same season/year

116, 202, 208, 209, 231, 233, 288-290 maybe elsewhere - haven’t introduced sites yet, I don’t have any context for these site name codes

134-136 does “distributed” vs “tower” plot type matter? They both end up being 20x20m plots, just in one case it is split between two subplots. 20x20m is 20x20m, I suggest that this may be extraneous information. If it isn’t be clearer as to why the different types matter.

138 you only give units for one of the measurements in this list of measurements. Give it for all or none. I suggest none, it is not important here.

159-169 you allude to it but the major shortcoming of hand delineation is that the delineator is working from the same dataset that the tree id algorithm is working from. Thus, it is not an independent truth dataset. You are actually comparing two ways of interpreting the same data. This can still be useful, but the comparison is fundamentally different than a comparison with field data. With field data you ask, how well does this compare to reality? With hand delineation from imagery you ask, how good of a job does the algorithm do given the information available in the images? Please flesh out a discussion of this distinction.

181-182 it is very important not to do this. It is good to separate an analysis based on trees you thought you could find vs trees you didn’t think you could find, but we can’t look at only the easy trees and then call that accuracy. This is similar to the last comment. You need to very carefully define what your benchmark is able to assess. If you remove the small trees, you have no way to assess accuracy of tree identification. Instead, you are assessing how well you can identify trees that you were pretty sure you should be able to identify. That might be a useful thing to do, but it is fundamentally different. Please include all trees in the dataset and flag the overstory trees so that both analyses can be completed. Please also improve your discussion of this topic.

222-223 This explanation of using bounding boxes comes way too late. Need to explain the rationale for your methods right up front. And your explanation is not satisfactory. Polygons are much more useful than bounding boxes. Give more reasoning and discuss the tradeoffs

198-199 “coordinates relative to top-left corner of image” extraneous information

238-251 this is an example of a writing style throughout this manuscript that is hard to read. You give the answer first (0.4 in this case), and then show your work backwards. It would be much easier to read if you were to put things in chronological order. For example, here you should start with the standard in CV literature, then talk about the tests you did, then talk about the number you chose. This manuscript would really benefit from a thorough revision to address backward exposition like this.

274-276 again, you shouldn’t leave the understory trees out here. We need to know how algorithms do in an absolute sense

279-283 it is mentioned several times that precision cannot be calculated as though that is the only shortcoming of this type of sampling. But scattered trees as ground truth has many more shortcomings. When the ground dataset is composed of scattered trees it is impossible to know whether the tree identification algorithm actually got the “right” tree, because you have no knowledge of the “wrong” trees. Just getting two boxes to overlap doesn’t necessarily mean that you actually got the right tree for the right reason. It is much more valuable to have small clusters of trees or full maps of contiguous areas. The shortcomings of this sampling need to be discussed in much more detail.

286 citation in different format than others

293-294 what is the smaller size or range of smaller sizes? Be specific.

339 what is a centroid rate?

359-361 you give this rationale in the caption and the paragraphs that refer to it, but you already described how you came up with the 0.4 number earlier and made no mention of this process. It is confusing for it to be described in two separate places without reference to one another. I can’t tell if this is actually the same threshold, or a different threshold that just happened to be the same number.

379 “repo” is not a word

395-396 this table caption is the first place you actually define recall and precision, despite using these terms throughout the manuscript. Definition should be at the first mention

Discussion: there are very few citations, even when discussing ideas and methods that warrant citation. For example on line 429, “regional proposal network” needs a citation

S1 explain the process of matching stem locations to bounding boxes in the main text, not in a supplement. Also, this method is quite weak. Calling something a match because a point is within a bounding box is very generous. If possible, improve this method using additional field-based measurements to validate the match. Also, is tree lean measured in the field and accounted for in mapping tree locations as points? If not, please express this as a shortcoming in the text.

S2 This is important information that belongs in the main text. It is also never cited, so the text frequently refer to sites that are never defined.

**********

**Have all data underlying the figures and results presented in the manuscript been provided?**

Large-scale datasets should be made available via a public repository as described in the *PLOS Computational Biology* data availability policy, and numerical data that underlies graphs or summary statistics should be provided in spreadsheet form as supporting information.

Reviewer #1: Yes

Reviewer #2: Yes

Reviewer #3: Yes

Reviewer #4: Yes

**********

PLOS authors have the option to publish the peer review history of their article (what does this mean?). If published, this will include your full peer review and any attached files.

If you choose “no”, your identity will remain anonymous but your review may still be made public.

**Do you want your identity to be public for this peer review?** For information about this choice, including consent withdrawal, please see our Privacy Policy.

Reviewer #1: **Yes: **Martí Bosch

Reviewer #2: **Yes: **Jonathan V. Williams

Reviewer #3: No

Reviewer #4: **Yes: **Sean M.A. Jeronimo

Figure Files:

While revising your submission, please upload your figure files to the Preflight Analysis and Conversion Engine (PACE) digital diagnostic tool, https://pacev2.apexcovantage.com. PACE helps ensure that figures meet PLOS requirements. To use PACE, you must first register as a user. Then, login and navigate to the UPLOAD tab, where you will find detailed instructions on how to use the tool. If you encounter any issues or have any questions when using PACE, please email us at figures@plos.org.

Data Requirements:

Please note that, as a condition of publication, PLOS' data policy requires that you make available all data used to draw the conclusions outlined in your manuscript. Data must be deposited in an appropriate repository, included within the body of the manuscript, or uploaded as supporting information. This includes all numerical values that were used to generate graphs, histograms etc.. For an example in PLOS Biology see here: http://www.plosbiology.org/article/info%3Adoi%2F10.1371%2Fjournal.pbio.1001908#s5.

Reproducibility:

To enhance the reproducibility of your results, PLOS recommends that you deposit laboratory protocols in protocols.io, where a protocol can be assigned its own identifier (DOI) such that it can be cited independently in the future. For instructions, please see http://journals.plos.org/compbiol/s/submission-guidelines#loc-materials-and-methods

Attachment Submitted filename: review 220221.docx Click here for additional data file.

# Author comment: Author response to Decision Letter 0

**DOI:** 10.1371/journal.pcbi.1009180.r002

13 May 2021

Attachment Submitted filename: Reviews Benchmark PLOS Computational Biology.docx Click here for additional data file.

# Peer review: Decision Letter 1

**DOI:** 10.1371/journal.pcbi.1009180.r003

**Authors:** Bjoern Peters, Jacopo Grilli

9 Jun 2021

Dear Dr. Weinstein,

We are pleased to inform you that your manuscript 'A benchmark dataset for canopy crown detection and delineation in co-registered airborne RGB, LiDAR and hyperspectral imagery from the National Ecological Observation Network' has been provisionally accepted for publication in PLOS Computational Biology.

Before your manuscript can be formally accepted you will need to complete some formatting changes, which you will receive in a follow up email. A member of our team will be in touch with a set of requests.

Please note that your manuscript will not be scheduled for publication until you have made the required changes, so a swift response is appreciated.

IMPORTANT: The editorial review process is now complete. PLOS will only permit corrections to spelling, formatting or significant scientific errors from this point onwards. Requests for major changes, or any which affect the scientific understanding of your work, will cause delays to the publication date of your manuscript.

Should you, your institution's press office or the journal office choose to press release your paper, you will automatically be opted out of early publication. We ask that you notify us now if you or your institution is planning to press release the article. All press must be co-ordinated with PLOS.

Thank you again for supporting Open Access publishing; we are looking forward to publishing your work in PLOS Computational Biology. 

Best regards,

Jacopo Grilli

Associate Editor

PLOS Computational Biology

Bjoern Peters

Benchmarking Editor

PLOS Computational Biology

***********************************************************

Reviewer's Responses to Questions

**Comments to the Authors:**

**Please note here if the review is uploaded as an attachment.**

Reviewer #3: A benchmark dataset for canopy crown detection and delineation in co-registered airborne RGB, LiDAR and hyperspectral imagery from the National Ecological Observation Network

I have reviewed the entire manuscript. The authors have substantially rewritten the manuscript in response to the previous review, and I believe that it should be published. I have a very few specific suggestions below that the authors may wish to consider.

Line 85: The wrong table is referenced. Table 2 is meant.

Line 118: The general rule of thumb for lidar delineation of crowns is >=8 pulses/m-2. You might extend the lower range to 8 instead of 20.

Line 233: The authors’ case for not including all field trees is weak. Users of the dataset should have full information on what trees are there and decide how to use it as relative to their goals. A field in this dataset could indicate whether a stem is likely to be an overstory tree based on the authors’ criteria.

Reviewer #4: The authors have done a good job responding to and incorporating comments. All comments were either addressed by adequate revisions or by adequate justification. The article is much easier to read. It clearly presents a tool that will be useful to many. I have only a few minor comments:

L67-70: This is a little confusing, since it says before this that you can do both tree detection (points) and crown delineation (polygons). Does this sentence refer only to the latter? If so, that isn't clear.

L93: Is the naming convention of the files an important detail to include?

L96-97, 105, 115-116, 131, 138, 147-148: I would prefer to see "see [23]" or "see [24]", etc., without the technical document ID, but that may be up to the journal style.

L122 Elsewhere you say 1000m x 1000m, here you say 1 km^2

L170 "types sites" looks like an error.

**********

**Have the authors made all data and (if applicable) computational code underlying the findings in their manuscript fully available?**

The PLOS Data policy requires authors to make all data and code underlying the findings described in their manuscript fully available without restriction, with rare exception (please refer to the Data Availability Statement in the manuscript PDF file). The data and code should be provided as part of the manuscript or its supporting information, or deposited to a public repository. For example, in addition to summary statistics, the data points behind means, medians and variance measures should be available. If there are restrictions on publicly sharing data or code —e.g. participant privacy or use of data from a third party—those must be specified.

Reviewer #3: Yes

Reviewer #4: Yes

**********

PLOS authors have the option to publish the peer review history of their article (what does this mean?). If published, this will include your full peer review and any attached files.

If you choose “no”, your identity will remain anonymous but your review may still be made public.

**Do you want your identity to be public for this peer review?** For information about this choice, including consent withdrawal, please see our Privacy Policy.

Reviewer #3: No

Reviewer #4: **Yes: **Sean MA Jeronimo

# Editor report: Acceptance letter

**DOI:** 10.1371/journal.pcbi.1009180.r004

**Authors:** Bjoern Peters, Jacopo Grilli

28 Jun 2021

PCOMPBIOL-D-20-01875R1 

A benchmark dataset for canopy crown detection and delineation in co-registered airborne RGB, LiDAR and hyperspectral imagery from the National Ecological Observation Network

Dear Dr Weinstein,

I am pleased to inform you that your manuscript has been formally accepted for publication in PLOS Computational Biology. Your manuscript is now with our production department and you will be notified of the publication date in due course.

The corresponding author will soon be receiving a typeset proof for review, to ensure errors have not been introduced during production. Please review the PDF proof of your manuscript carefully, as this is the last chance to correct any errors. Please note that major changes, or those which affect the scientific understanding of the work, will likely cause delays to the publication date of your manuscript. 

Soon after your final files are uploaded, unless you have opted out, the early version of your manuscript will be published online. The date of the early version will be your article's publication date. The final article will be published to the same URL, and all versions of the paper will be accessible to readers.

Thank you again for supporting PLOS Computational Biology and open-access publishing. We are looking forward to publishing your work! 

With kind regards,

Zsofi Zombor

PLOS Computational Biology | Carlyle House, Carlyle Road, Cambridge CB4 3DN | United Kingdom ploscompbiol@plos.org | Phone +44 (0) 1223-442824 | ploscompbiol.org | @PLOSCompBiol
